# Aqueous Carbonation of Calcium Silicates With Different Ca/Si Ratios Studied by Solid‐State NMR Spectroscopy

**DOI:** 10.1002/mrc.5528

**Published:** 2025-05-15

**Authors:** Rune Wittendorff Mønster Jensen, Jørgen Skibsted

**Affiliations:** ^1^ Department of Chemistry and Interdisciplinary Nanoscience Center (iNANO) Aarhus University Aarhus C Denmark

**Keywords:** ^13^C, ^29^Si, CaCO_3_, calcium silicates, carbonation, CO_2_ sequestration, hydration, NMR, silica gel

## Abstract

Calcium silicates react readily with CO_2_ under aqueous conditions, forming CaCO_3_ and silica gel. This is utilized to produce new cement binders and to sequester CO_2_, thereby contributing to a lowering of the CO_2_ footprint for the cement industry. The present work investigates aqueous carbonation of three hydraulic and three non‐hydraulic calcium silicates with the aim of analyzing the impact of the Ca/Si ratio on the structure of the amorphous silica gel and on the extent and rate of carbonation. This information is obtained from ^29^Si NMR experiments, whereas ^13^C NMR and FT‐IR are used to characterize the polymorphic forms of CaCO_3_ formed upon carbonation. The structure of the silica gel is not dependent on the type of carbonated calcium silicate or their Ca/Si ratio. In addition, the amounts of CaCO_3_ from TGA analysis match well the theoretical maximum values. ^29^Si and ^29^Si{^1^H} CP/MAS spectra of a commercial silica gel are very similar to those observed for the carbonated calcium silicates, which strongly suggests that a hydroxylated silica gel without incorporated Ca ions constitutes the silica gel in carbonated calcium silicates. From ^13^C NMR and FT‐IR, it is found that calcite is the principal CaCO_3_ polymorph for all samples carbonated for 6 h. However, aragonite and calcite do co‐exist during the initial carbonation (20 min) of γ‐Ca_2_SiO_4_. Comparison of the carbonation evolution for the hydraulic and non‐hydraulic calcium silicates strongly suggests that an early hydration and formation of C‐S‐H is not a required initial step in the aqueous carbonation process.

## Graphical abstract

Six calcium silicates with Ca/Si ratios from 1 to 3 have been exposed to aqueous carbonation at ambient conditions and characterized by ^29^Si and ^13^C MAS and CP/MAS NMR spectroscopy. The analysis reveals that the structure and composition of the produced silica gel is independent of the starting materials Ca/Si ratio. Furthermore, it is shown that a hydrated phase is not an initial step of the carbonation process, and that hydraulic and non‐hydraulic calcium silicates exhibit very similar carbonation kinetics.

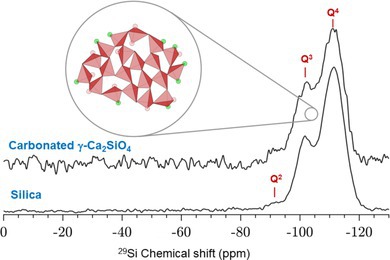

## 1 Introduction

Concrete represents the principal construction material in the world, reflecting its wide applications in our society for infrastructure such as roads, tunnels, bridges, and housing. Hydrated cement constitutes the binder in concrete, and approx. 4 billion tons Portland cement is produced annually worldwide [1]. This large amount results in that approx. 7% of the anthropogenic CO_2_ emissions originate from cement production [2]. The CO_2_ emissions from Portland cement production originate mainly from decalcification of limestone (CaCO_3_) and from the fuels for heating the cement kiln and for milling, accounting for roughly 60% and 40% of the released CO_2_, respectively. Thus, the production of 1 t of Portland cement releases about 800 kg of CO_2_ [3].

The principal components of Portland cement are calcium silicates (i.e., alite‐Ca_3_SiO_5_ and belite‐β‐Ca_2_SiO_4_), tricalcium aluminate (Ca_3_Al_2_O_6_), calcium aluminoferrite (Ca_2_(Al_2‐x_Fe_x_)O_5_), and gypsum (CaSO_4_·2H_2_O). These phases react with water and form principally a less‐ordered aluminum‐substituted calcium‐silicate‐hydrate (C‐(A)‐S‐H) phase, the “glue in concrete”, and smaller amounts of portlandite (Ca(OH)_2_) and the calcium aluminate hydrate phases ettringite (Ca_6_Al_2_(OH)_12_(SO_4_)_3_·26H_2_O) and monosulfate (Ca_4_Al_2_(OH)_12_(SO_4_)·6H_2_O). During the service life of concrete, which can extend over 100 years, these hydrates partially decompose as a result of reactions with atmospheric CO_2_ [4] and other anions such as chloride and sulfate ions, if exposed to for example surface water or seawater. Atmospheric CO_2_ reacts with water, forming carbonate ions that in solution will react with the principal cement hydration products, forming calcium carbonate and silica or alumina‐silica gel phases. This implies that over the life span of a concrete construction, a significant amount of CO_2_ from the atmosphere can be bound chemically in the material before it disintegrates [5]. Thus, concrete acts as a sink for CO_2_.

Currently, carbonation of cementitious materials is being extensively explored by academic and industrial researchers in the cement community, as end‐of‐life concrete and Ca‐bearing waste materials have a huge potential for binding large amounts of CO_2_ if they are exposed to enforced carbonation processes. For example, recent studies of hydrated Portland cement pastes exposed to aqueous carbonation at ambient conditions have shown that nearly all Ca‐bearing phases in hydrated cement pastes can be carbonated, forming CaCO_3_ and an amorphous alumina‐silica gel as the main carbonation products [6]. Thereby 100 g of cement paste can bind up to 45 g of CO_2_ [7], illustrating the huge potential of utilizing end‐of‐life concrete as a global sink for CO_2_ sequestration.

Enforced carbonation and carbonation curing of anhydrous cements and Ca‐bearing waste materials can also be utilized in hardening processes to produce new types of binders [8] with the potential of strongly reducing the CO_2_ footprint relative to Portland cement binders. This has been explored in carbonation processes for a number of pure calcium silicate phases [9, 10, 11, 12, 13, 14, 15, 16, 17, 18, 19, 20, 21, 22, 23, 24, 25, 26], for example utilizing the reactive hydrothermal liquid phase densification (rHLPD) process [27], to produce precast elements. The calcium silicates have Ca/Si ratios ranging from 3 (tricalcium silicate or alite, Ca_3_SiO_5_ = C_3_S) to 1 (wollastonite, CaSiO_3_ = CS), and a shorthand cement notation is often used where C = CaO, S = SiO_2_, and H = H_2_O. Moreover, they can be divided into two groups depending on their reaction with water at ambient conditions. The hydraulic calcium silicates include tricalcium silicate in its triclinic and monoclinic form (T‐C_3_S and M‐C_3_S) as well as belite (β‐C_2_S) whereas γ‐C_2_S, rankinite (C_3_S_2_) and wollastonite (CS) are non‐hydraulic phases. Generally, the calcium silicates are produced by heating stoichiometric amounts of limestone and sand (silica or quartz) at temperatures from 900°C to 1500°C, according to the reaction:

(1) $$x\;\mathrm{CaC}O_{3}\left(s\right)+\mathrm{Si}O_{2}\left(s\right)\overset{\Delta }{\to }{\left(\mathrm{CaO}\right)}_{x}\mathrm{Si}O_{2}\left(s\right)+x\;CO_{2}\left(g\right)$$

This equation highlights the significant release of CO_2_ associated with the production of M‐C_3_S and β‐C_2_S, which are the principal components of Portland cement. Upon hydration, these phases form a less‐crystalline calcium‐silicate‐hydrate (C‐S‐H) phase, with variable composition, i.e., Ca/Si = 0.8–2.0 with a mean of ~1.75 [28] and H_2_O/Si ≈ 3 [29], and additionally portlandite (Ca(OH)_2_):

(2) $$\begin{array}{c} {\left(\mathrm{CaO}\right)}_{x}\mathrm{Si}O_{2}\left(s\right)+z\;H_{2}O\left(l\right)\to {\left(\mathrm{CaO}\right)}_{x-y}\mathrm{Si}O_{2}{\left(H_{2}O\right)}_{z-y}\left(s\right)+y\;\mathrm{Ca}{\left(\mathrm{OH}\right)}_{2}\left(s\right) \end{array}$$

All of the abovementioned calcium silicates can be carbonated under aqueous or humid conditions, and the process is a dissolution–precipitation reaction, where dissolved Ca^2+^ ions react with carbonate ions in solution, forming calcium carbonate and a silica gel [6], following the equation:

(3) $$\begin{array}{c} {\left(\mathrm{CaO}\right)}_{x}\mathrm{Si}O_{2}\left(s\right)+a\;CO_{2}\left(\mathrm{aq}\right)\to a\;\mathrm{CaC}O_{3}\left(s\right)+{\left(\mathrm{CaO}\right)}_{x-a}\mathrm{Si}O_{2}\left(s\right) \end{array}$$

Under ideal conditions, a = x, corresponding to the maximum amount of CaCO_3_ and the formation of a pure silica gel. Pioneering research on wet carbonation of calcium silicates and calcium silicate cements, performed by Ashraf, Olek and co‐workers [15, 30, 31, 32, 33], has shown that the silica gel may also contain minor amounts of calcium ions, depending on the composition (Ca/Si ratio) and carbonation conditions. They also reported that the formed silica gel contains a small amount of water or hydroxyl groups, which may be in the range H_2_O/Si ≈ 0.1–0.6, depending on the carbonation conditions and extent of carbonation. The carbonation of the C_3_S, C_2_S, and C_3_S_2_ phases occurs readily at ambient conditions and can continue to their completion, depending on the actual conditions. However, for wollastonite (CS) elevated temperatures are required to obtain a high conversion degree [5]. The carbonation of calcium silicates under aqueous conditions may result in an initial formation of a C‐S‐H phase and portlandite (Equation (2)). The produced portlandite reacts readily with carbonate ions and may initially consume most of the carbonate ions in solution. Subsequently, the carbonation of the C‐S‐H phase takes place, firstly by a decalcification, where interlayer Ca ions are removed from the structure, and secondly by decomposition of the low‐Ca/Si ratio C‐S‐H into CaCO_3_ and SiO_2_ [34, 35, 36]. Thus, it has been speculated in several studies whether hydration of the calcium silicates is the first step in carbonation process.

The carbonation processes for calcium silicates have been studied in great detail, providing information about reaction kinetics and reaction products as a function of physical parameters such as temperature, CO_2_ concentration and gas flow, relative humidity, and state of water in the process. These investigations have utilized a variety of experimental methods, including thermogravimetric analysis (TGA), powder X‐ray diffraction (XRD), Fourier‐transformed infrared spectroscopy (FT‐IR), solid‐state NMR spectroscopy, along with microscopic tools such as scanning electron microscopy (SEM) analysis [9, 10, 11, 12, 13, 14, 15, 16, 17, 19, 20, 21, 22, 23, 24, 25, 26]. Of these techniques, solid‐state ^27^Al and ^29^Si magic‐angle spinning (MAS) NMR experiments have been particularly informative, since these methods allow detection of crystalline as well as amorphous phases in an equal manner. Solid‐state NMR is widely used in studies of Portland cement hydration [37, 38], as the principal hydration product is a less‐ordered aluminum‐substituted C‐S‐H phase [39, 40] and since several blended cements contain amorphous supplementary cementitious materials (SCM's) such as fly ashes, slags, silica fume and calcined clays [41]. In carbonation of calcium silicates and Portland cement paste, ^27^Al and ^29^Si NMR have provided unique information about the decalcification and decomposition of the C‐S‐H phase and the associated formation of the (alumina) silica gel [7, 32, 34]. The latter product exhibits an amorphous structure including SiO_4_ tetrahedra in different coordination states, e.g., Q^3^ and Q^4^ sites, and the formation of these and their relative amounts can easily be determined by ^29^Si NMR. In a recent study of aqueous carbonation of Portland cement pastes including different types of SCM's, it was possible to identify different types of Q^3^(nAl) and Q^4^(mAl) sites by a combination of ^27^Al and ^29^Si MAS NMR and ^29^Si{^1^H} CP/MAS NMR experiments, where the quantification of these sites resulted in a model for the structure of the alumina‐silica gel which accounts for its Al/Si ratio and number of non‐bonded oxygens [42].

In the present work, we investigate the aqueous carbonation of three hydraulic and three non‐hydraulic calcium silicates with focus on how the Ca/Si ratio of the starting material and its hydraulic properties affect reaction rates, intermediate phases, and the structure of the amorphous silica gel. The principal tools are single‐pulse ^29^Si MAS and ^29^Si{^1^H} CP/MAS experiments, however, ^13^C MAS and ^13^C{^1^H] CP/MAS NMR are also used to study the formation of CaCO_3_ and its specific polymorphic forms during carbonation. The solid‐state NMR studies are supplemented with data from TGA and FT‐IR analyses, which are used to validate the degrees of carbonation and the formed carbonation products.

## 2 Materials and Methods

### 2.1 Materials

Six synthetic calcium silicates with different calcium‐to‐silicon ratios and representing hydraulic (T‐C_3_S, M‐C_3_S and β‐C_2_S) and non‐hydraulic (γ‐C_2_S, C_3_S_2_ and CS) phases have been studied in this work. All samples, except for CS (wollastonite), were purchased from VUSTAH, Czech Republic, as powdered samples and used as received. The CS sample has been prepared in an earlier project conducted in our laboratory [43]. The basic purity of the calcium silicates were confirmed by powder X‐ray diffraction, ^29^Si MAS NMR and TGA analyses. The latter method provided the content of CaCO_3_ in the samples, giving the following values: 1.2 wt%—T‐C_3_S, 5.2 wt%—M‐C_3_S, 1.2 wt%—β‐C_2_S, 4.8 wt%—γ‐C_2_S, and 3.0 wt%—CS. The ^29^Si NMR spectra reveal that the samples are phase pure, except for γ‐C_2_S which contains 8.3 wt.% β‐C_2_S and 86.9 wt% γ‐C_2_S according to the peak intensities in the ^29^Si NMR spectra and the impurity of CaCO_3_. The samples were ground to a particle size below 45 μm.

### 2.2 Carbonation Procedures

The enforced aqueous carbonation of the samples employed an experimental setup developed in a recent project in our laboratory [42], following the approach by Zajac et al. [44]. The carbonation was conducted in 30 mL deionized water exposed to a continuous flow of 10% CO_2_ (1 L/h CO_2_) and 90% N_2_ (9 L/h N_2_). When equilibrium between the gas and solution was achieved, 0.50 g of the calcium silicate sample was added to the solution under stirring with a magnetic bar at 600 rpm. Standard carbonation experiments under these conditions were performed for 6 h at ambient temperature (~20°C). An exception is the CS sample for which the carbonation was conducted at elevated temperature (60°C) and for 24 h, following reported data in literature [12, 15, 18, 19]. The experiments were stopped at selected carbonation times (5 min to 6 h) at which the solids were isolated by suction filtration and dried in a desiccator for at least 5 days before further characterization.

### 2.3 Characterization Procedures

The solid‐state ^29^Si NMR experiments were conducted at 79.49 MHz on a Bruker Avance 400 MHz NMR spectrometer (9.4 T) using a 4 mm Bruker X{^1^H/^19^F} CP/MAS probe. The single‐pulse ^29^Si NMR spectra were recorded with a spinning frequency of 10.0 kHz, a 90° excitation pulse of 4.0 μs (γB_1_/2π = 62.5 kHz), a recycle delay of 120 s, and 512–1024 scans. The effect of ^29^Si spin–lattice relaxation was examined for selected calcium silicates after carbonation for 6 h by acquisition of arrays of spectra with increasing relaxation delays. Although full relaxation is not completely obtained with a relaxation delay of 120 s, this value was chosen as a compromise to balance signal‐to‐noise ratio and instrument time (17–34 h per spectrum). This is justified by simulations of spectra acquired with relaxation delays of 120 and 240 s, which revealed nearly identical relative intensities. The spectra were referenced to tetramethylsilane (TMS) using a sample of β‐C_2_S as a secondary reference (δ_iso_ = −71.33 ppm). The ^29^Si{^1^H} CP/MAS experiments were acquired with a spinning frequency of 5.0 kHz and a ^1^H‐ramped CP sequence, using a 90° ^1^H excitation pulse of 2.4 μs (γB_2_/2π = 104 kHz) and optimized spin‐lock pulses of γ_Si_B_1Si_/2π = 62.5 kHz and γ_H_B_1H_/2π = 72.8–104 kHz, with a CP contact time of 3.0 ms. In addition, a relaxation delay of 4.0 s was used, and 4096–65,536 scans were accumulated.

Simulations of the single‐pulse ^29^Si MAS and ^29^Si{^1^H} CP/MAS spectra were performed with the Bruker TopSpin 3.6.4 software. The optimum result was obtained by a Gaussian/Lorentzian model for the resonance line shapes, using pure Lorentzian line shapes for unreacted calcium silicates, a 1:1 mixture of Gaussian/Lorentzian line shapes for the Q^1^/Q^2^ resonances, and Gaussian line shapes for the Q^3^/Q^4^ resonances.

The solid‐state ^13^C and ^13^C{^1^H} CP/MAS NMR spectra were recorded at 75.30 MHz on a Varian INOVA 300 MHz (7.05 T) spectrometer using a 7‐mm home‐built CP/MAS probe. The single‐pulse ^13^C NMR spectra were recorded with a spinning frequency of 4.0 kHz, a 90° excitation pulse of 7.1 μs (γB_1_/2π = 35 kHz), a recycle delay of 500 s, and 128–512 scans. The ^13^C{^1^H} CP/MAS spectra also employed ν_R_ = 4.0 kHz and were recorded with a 90° ^1^H excitation pulse of a 4.75 μs (γB_2_/2π = 52 kHz) and optimized spin‐lock field strengths of γ_H_B_1H_/2π = 26–31 kHz and γ_C_B_1C_/2π = 35 kHz. The spectra employed a CP contact time of 4.0 ms, a relaxation delay of 4 s, and 3840–15,360 scans. All spectra were processed with 50 Hz apodization and were referenced to TMS, using the methyl groups of hexamethylbenzene as a secondary reference (δ_iso_ = 17.3 ppm).

The ^13^C and ^29^Si NMR spectra shown in the figures have been normalized to the actual sample amounts in the NMR rotors and the number of scans acquired for the individual spectra.

TGA data were measured on a NETZCH TG 209 Libra instrument. About 20–30 mg of sample were heated from 30°C to 980°C in open Al_2_O_3_ crucibles, with a heating rate of 20°C/min and 20 mL/min N_2_ purge gas. The TGA curves were analyzed by assigning all events above 300°C to the release of CO_2_, and mass losses below 300°C to the liberation of H_2_O.

ATR FT‐IR measurements were recorded on a PerkinElmer spectrum Two FT‐IR spectrometer. The spectra were measured with a spectral range from 400 to 4000 cm^−1^ and with a 1.0 cm^−1^ resolution.

## 3 Results and Discussion

The results are presented and discussed in five subsections. Section 3.1 focusses on the structure of silica gel formed for the different calcium silicates at their maximum achieved degrees of carbonation. Section 3.2 considers the amounts CaCO_3_ formed for the calcium silicate samples and is based on TGA measurements. The formation of CaCO_3_ and its polymorphic forms are investigated by FT‐IR and ^13^C NMR experiments in Sections 3.3 and 3.4, respectively. Finally, Section 3.5 examines the rate of carbonation for four of the studied calcium silicates.

### 3.1 Structure of the Silica Gel

**FIGURE 1:**
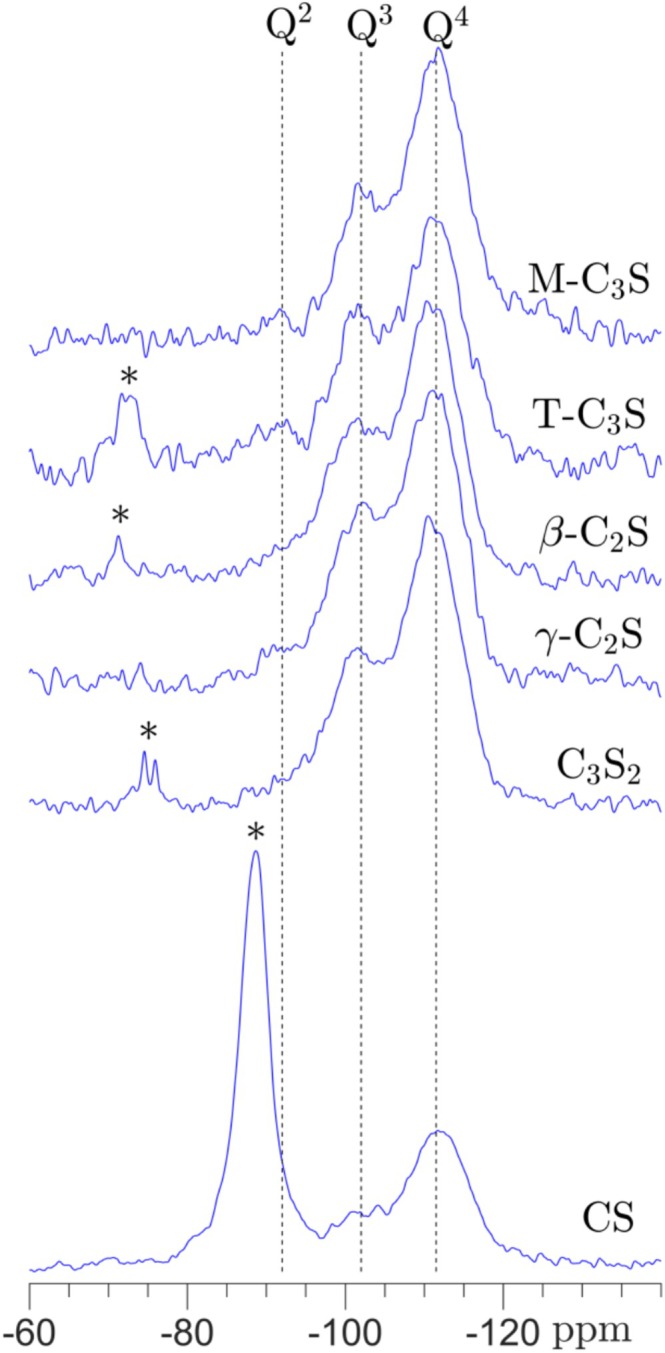
Single‐pulse ^29^Si NMR spectra (9.4 T) of the carbonated calcium silicates acquired with a spinning frequency of 10.0 kHz and a relaxation delay of 120 s. All samples, except for CS, have been carbonated at ambient temperature (20°C) for 6 h. The spectrum of CS corresponds to a sample carbonated at 60°C for 24 h. Resonances from unreacted calcium silicates are indicated by asterisks.

The six studied calcium silicates have been carbonated in water at ambient conditions for 6 h, and single‐pulse ^29^Si NMR spectra of the carbonation products are shown in Figure 1. The spectra reveal a full degree of carbonation for M‐C_3_S and γ‐C_2_S, whereas remains of unreacted material are seen for the four other calcium silicates. Integration over the resonances in these spectra gives degrees of carbonation of 94%, 97%, and 98% for T‐C_3_S and β‐C_2_S and C_3_S_2_, respectively, demonstrating high degrees of carbonation for these samples as well. However, the ^29^Si NMR spectrum of wollastonite, carbonated under ambient conditions mentioned above (not shown), showed no indications of any carbonation at all. Thus, an experiment was conducted for CS where the temperature in the reaction cell was increased to 60°C and the carbonation time was extended to 24 h. The ^29^Si NMR spectrum of this sample is included in Figure 1, where integration over the peaks gives a carbonation degree of 42%. This lower degree of carbonation for CS is in agreement with earlier studies, which have shown that elevated temperatures, high CO_2_ concentrations, and significantly longer exposure times are needed to obtain a high degree of carbonation for CS [12, 15, 18, 19].

**FIGURE 2:**
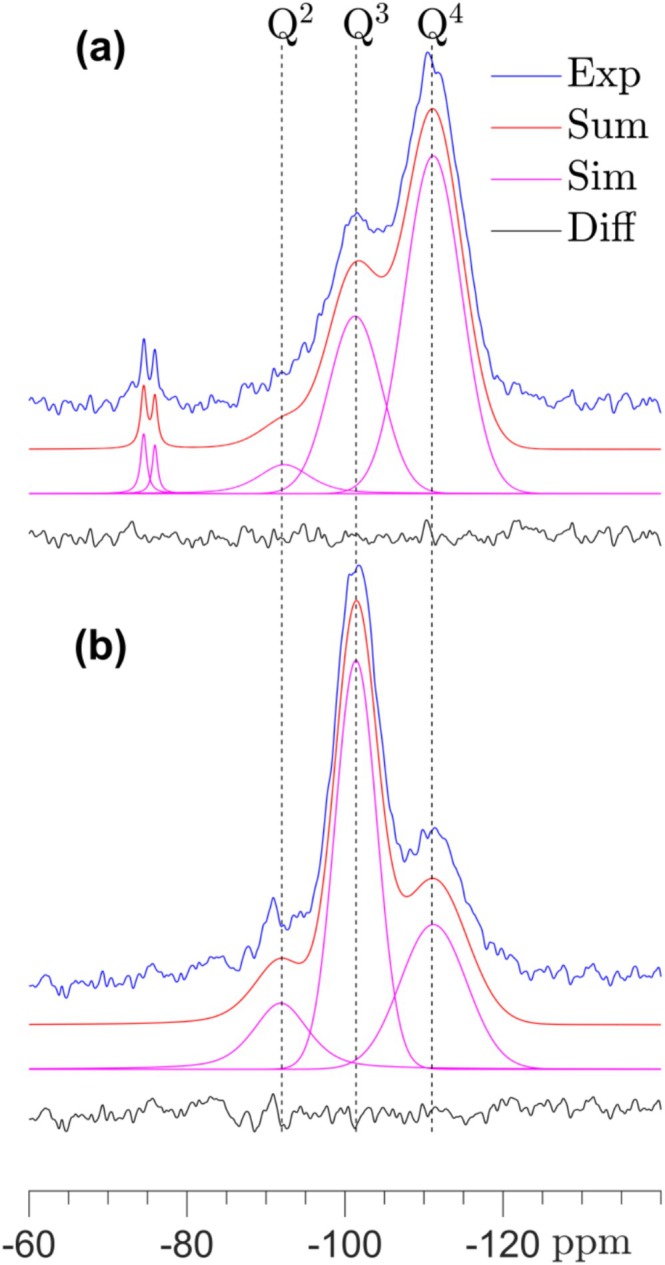
Experimental and simulated single‐pulse ^29^Si MAS and ^29^Si{^1^H} CP/MAS NMR spectra (9.4 T) of rankinite (C_3_S_2_) carbonated for 6 h at ambient conditions. The single‐pulse spectrum is acquired with a spinning frequency of ν_R_ = 10.0 kHz and a relaxation delay of 120 s, whereas the CP spectrum employs ν_R_ = 5.0 kHz, a 4 s relaxation delay, and a CP contact time of τ_CP_ = 3.0 ms.

**FIGURE 3:**
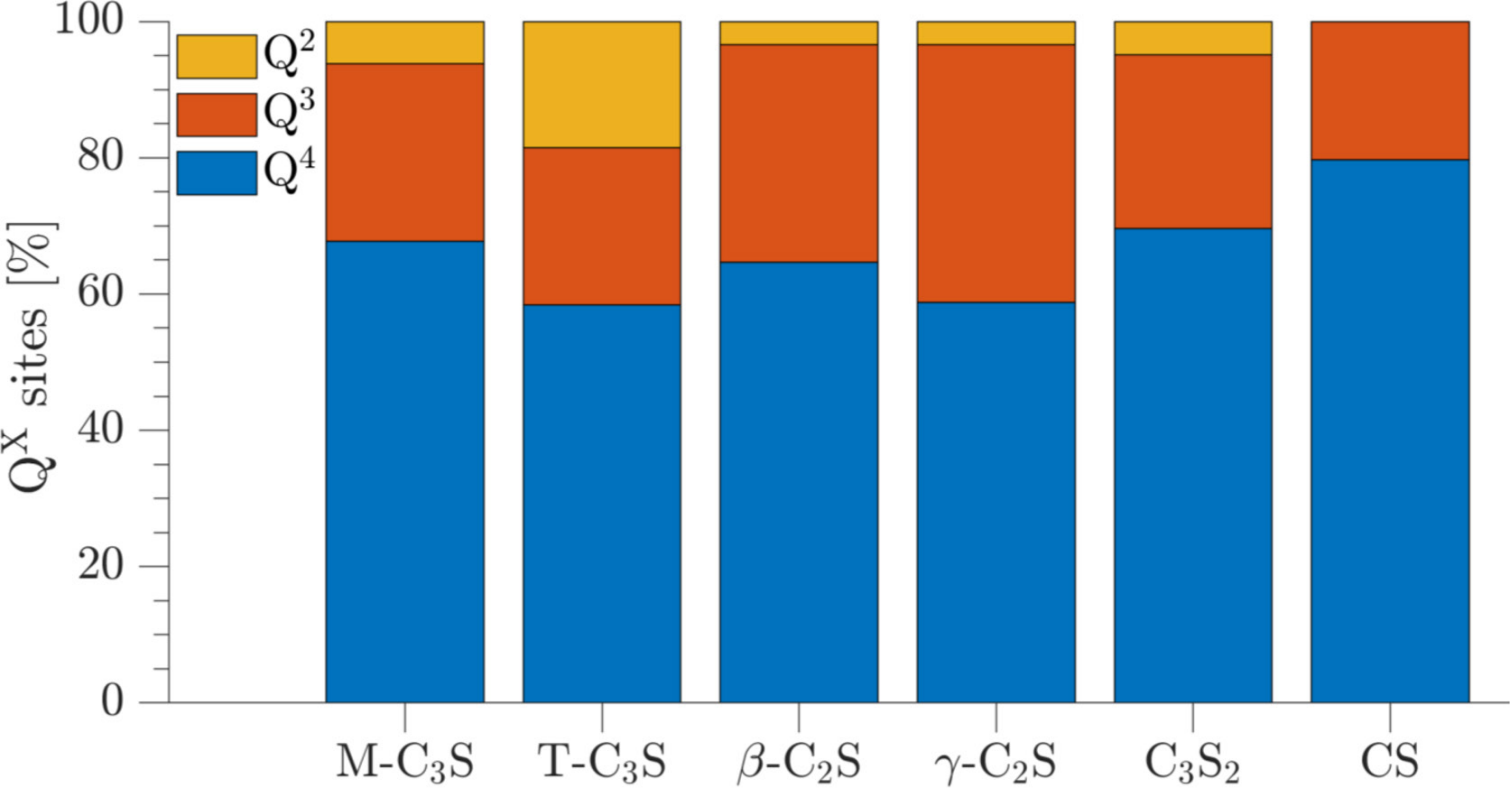
Fractions of Q^2^, Q^3^, and Q^4^ sites of the silica gels produced by carbonation of the six calcium silicates. The fractions are derived from simulations of the ^29^Si NMR spectra in Figure 1.

The amorphous silica gel, resulting from carbonation of the calcium silicates, are in all spectra seen by broadened resonances at −92, −102, and −112 ppm, corresponding to Q^2^, Q^3^, and Q^4^ types of SiO_4_ tetrahedra. No resonances seem present in the range −78 to −85 ppm in the single‐pulse ^29^Si NMR spectra, indicating that a calcium‐silicate‐hydrate (C‐S‐H) phase, the principal hydration product for hydraulic calcium silicates, is not present in the samples after carbonation for 6 h. Thus, the ^29^Si NMR spectra have been simulated using three resonances for the silica gel (i.e., Q^2^, Q^3^, and Q^4^) and additional peaks for the unreacted components, as illustrated in Figure 2 for C_3_S_2_. The relative intensities, resulting from these simulations, are shown in Figure 3 for the six calcium silicates, normalized to 100% for the Q^2^, Q^3^, and Q^4^ peaks. The silica gel is dominated by fully condensed SiO_4_ tetrahedra for all calcium silicates with a very similar fraction of Q^4^ sites (58–64%) for all samples, excluding carbonated CS. This indicates that the structure of the silica gel is not dependent on the type of carbonated calcium silicate or their Ca/Si ratio. The intensities in Figure 3 show also that the fraction of Q^2^ sites is higher in the C_3_S samples as compared to C_2_S and C_3_S_2_.

**FIGURE 4:**
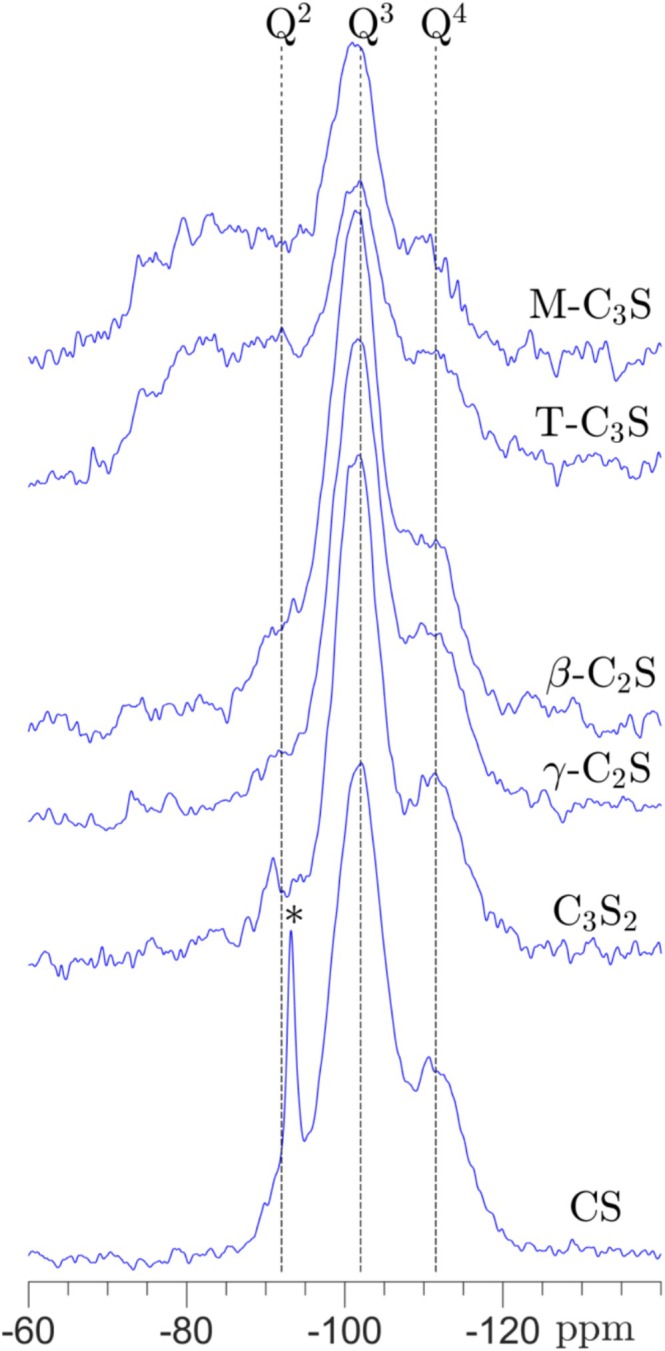
^29^Si{^1^H} CP/MAS NMR spectra (9.4 T) of the calcium silicates carbonated at ambient temperature for 6 h, except for CS which was carbonated at 60°C for 24 h. The spectra are obtained with ν_R_ = 5.0 kHz, a 4 s relaxation delay, and a CP contact time of τ_CP_ = 3.0 ms. The asterisk indicates the resonance from an impurity in the original sample of CS before carbonation.

The non‐bridging oxygens of the Q^2^ and Q^3^ sites may originate from hydroxyl groups, present at the surface of the silica gel. However, it has also been proposed that non‐bridging oxygens can be stabilized/charge‐balanced by Ca^2+^ ions cross‐linking Q^2^ or Q^3^ tetrahedra in the gel structure [13, 15, 32, 34, 45]. To examine this in more detail, the carbonated samples have been analyzed by ^29^Si{^1^H} CP/MAS NMR (Figure 4), which selectively detects silicate species with hydrogen atoms in their near vicinity. These spectra show a clear intensity enhancement for the Q^3^ sites, reflecting that these are hydroxylated SiO_4_ sites, i.e., HO‐Si*(‐O‐Si)_3_ sites. The Q^4^ sites are also clearly observed, and their presence in the spectra originate from Q^4^ sites sharing an oxygen atom with a hydroxylated Q^3^ site, i.e., HO‐Si‐O‐Si*‐(O‐Si)_3_ sites. Q^2^ sites, with lower intensities and at roughly −92 ppm, are also observed for the CS, C_3_S_2_, and the two C_2_S polymorphs, which are ascribed to the presence of (HO‐)_2_Si*(‐O‐Si)_2_ sites. The ^1^H → ^29^Si cross‐polarization efficiency for these sites may be stronger than for the Q^3^ sites, as they have two hydrogens in their second coordination sphere. Thus, the fraction of Q^2^ sites may be lower than indicated by the intensities in Figure 4. Broad resonances in the approximate range −72 to −94 ppm are observed for both M‐C_3_S and T‐C_3_S, showing the presence of hydrated or hydroxylated Q^0^, Q^1^, and Q^2^ sites. These sites may originate from hydroxylated silicon sites of a C‐S‐H phase, formed along with the silica gel, which has not been fully carbonated after 6 h. Further information on the mutual presence of the C‐S‐H and silica gel is derived from the ^29^Si and ^29^Si{^1^H} CP/MAS NMR spectra following the time evolution of the carbonation process in detail for T‐C_3_S, β‐C_2_S, γ‐C_2_S, and C_3_S_2_ (vide infra).

**FIGURE 5:**
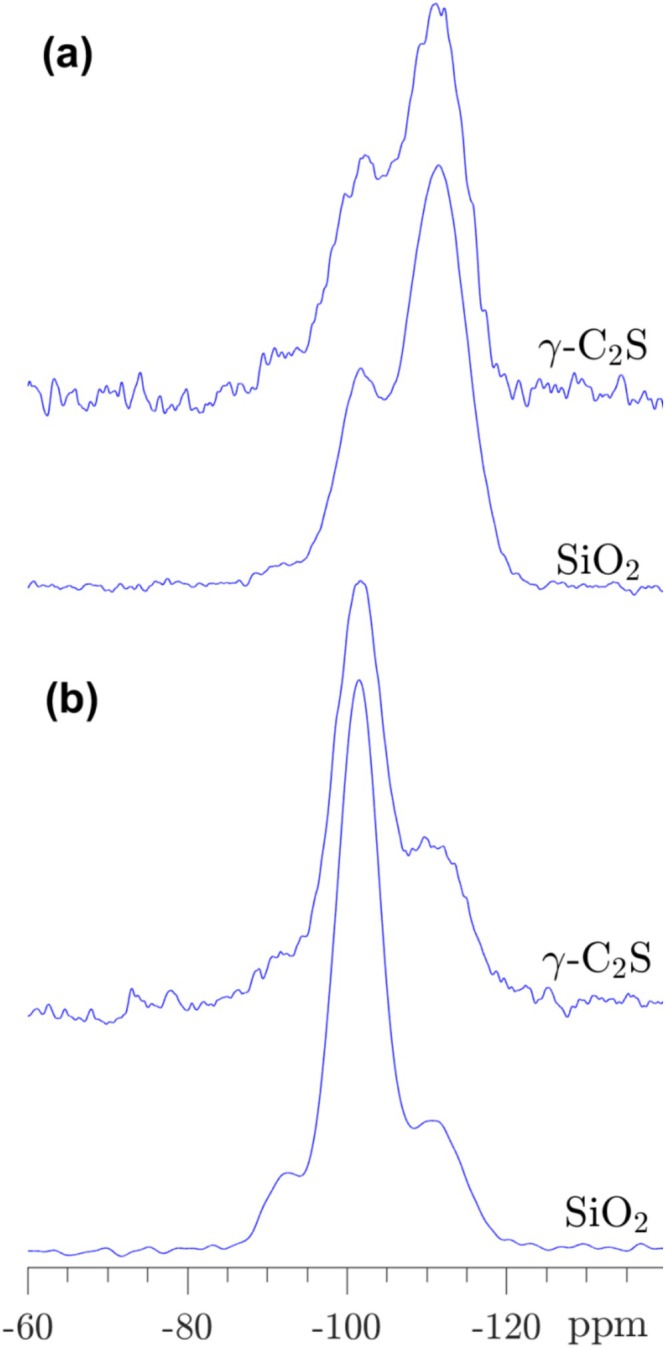
(a) Single‐pulse ^29^Si MAS and (b) ^29^Si{^1^H} CP/MAS NMR spectra (9.4 T) of carbonated γ‐C_2_S (6 h at ambient temperature) and a commercial silica gel (Sigma‐Aldrich, 230–400 mesh). The spectra in (a) are obtained with ν_R_ = 10.0 kHz and a 120 s relaxation delay, whereas the CP/MAS NMR spectra (b) employed ν_R_ = 5.0 kHz, a 4 s relaxation delay, and a CP contact time of τ_CP_ = 3.0 ms.

Comparison of the resonances from the silica gel in the ^29^Si MAS and ^29^Si{^1^H} CP/MAS spectra reveal only minor intensity variations for the Q^2^, Q^3^, and Q^4^ sites (Figures 2, 3, 4), suggesting that its structure and composition is independent on the composition (Ca/Si) of the calcium silicates before carbonation. Moreover, the resonances are very similar to those observed for a commercial silica gel, as illustrated in Figure 5 by comparison of ^29^Si MAS and ^29^Si{^1^H} CP/MAS spectra for carbonated γ‐C_2_S and a commercial silica gel (Sigma‐Aldrich, 230–400 mesh). Simulation of the ^29^Si NMR spectrum for the commercial SiO_2_ gel gives relative intensity fractions of Q^2^ = 4.7%, Q^3^ = 24.9%, and Q^4^ = 70.4%, which corresponds to a lower Q^3^/Q^4^ ratio (0.35) as compared to the ratio for the silica gel of carbonated γ‐C_2_S (Q^3^/Q^4^ = 0.64). This may reflect that the particles of silica gel in the carbonated sample of γ‐C_2_S are slightly smaller than those of the commercial SiO_2_ gel with particle sizes in the range 40–63 μm. This finding is further supported by the ^29^Si{^1^H} CP/MAS NMR spectra (Figure 5b), since a higher fraction of Q^4^ sites are observed for the gel of carbonated γ‐C_2_S as compared to the commercial SiO_2_ gel, corresponding to a higher fraction of Q^4^ sites close to the surface of the silica particles. The commercial gel contains water molecules and hydroxyl groups on the surface of the particles, as seen by TGA analysis which shows mass losses of 5.7 wt% from 20°C–200°C and of 3.0 wt% in the range 300°C–750°C, corresponding to the loss of water molecules and hydroxyl groups, respectively. These values give the approximate composition, SiO_2_·0.32H_2_O, for the commercial silica gel, and a OH^−^/Si ratio of 0.22. This ratio is lower than NBO/Si = 0.34, calculated from the ^29^Si NMR intensities, which implies that only 0.22/0.34 of the non‐bridging oxygens (NBOs) are hydroxylated, where the remaining NBO's may interact with water molecules on the surface via hydrogen bonding. If the silica gel from γ‐C_2_S carbonation has a similar hydrous/hydroxylated surface structure as the commercial gel, its higher NBO/Si = 0.45 would suggest a lower coverage of NBO's by hydroxyl groups. This may indicate that Ca^2+^ ions contribute to charge‐balancing the NBO's, for example by cross‐linking of Q^3^ tetrahedra, i.e., (O‐)_3_Si‐O⋯Ca⋯O‐Si(‐O_3_) sites.

### 3.2 Amounts of CaCO_3_ From Carbonation Experiments

**FIGURE 6:**
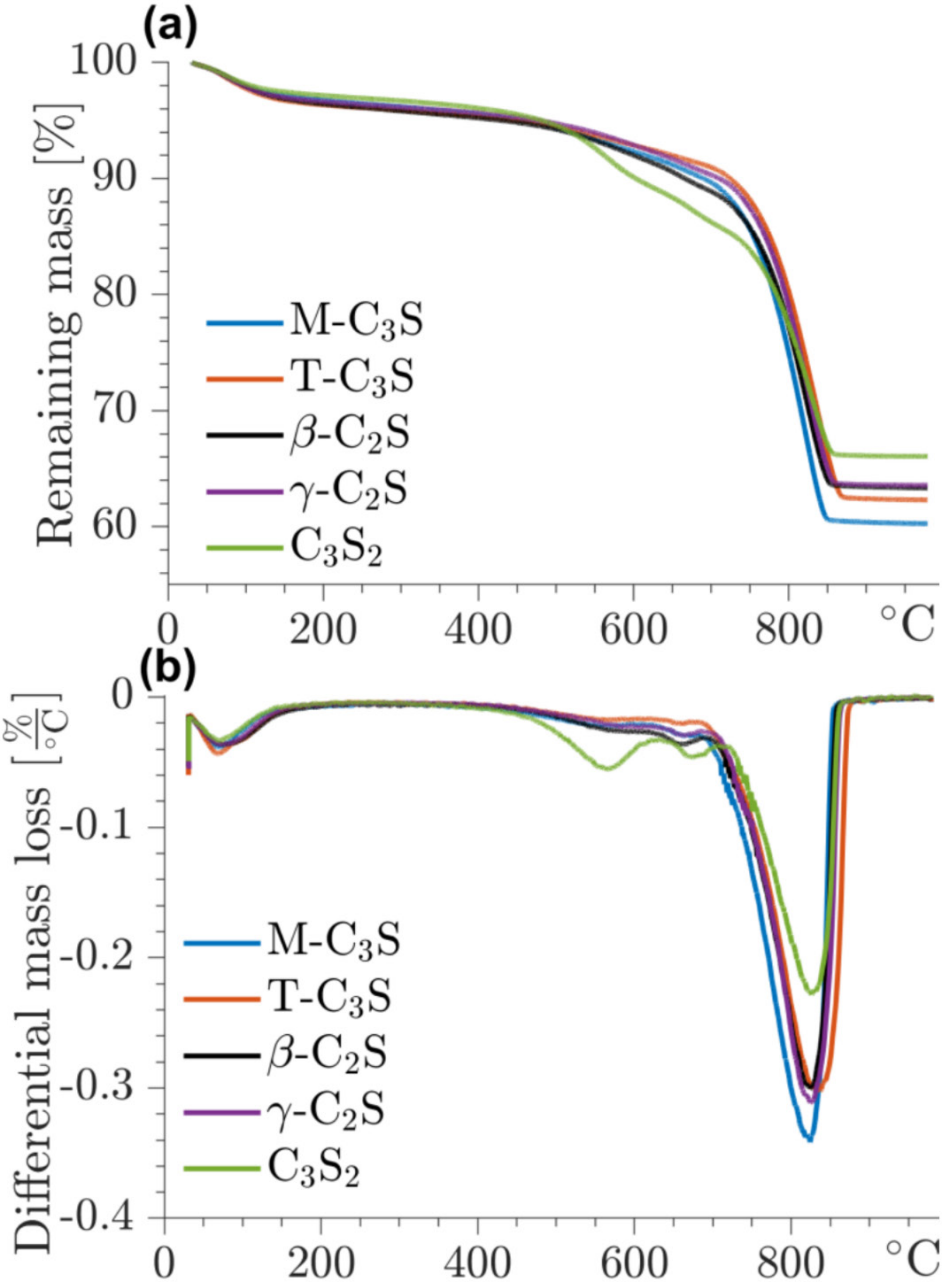
Stacked (a) TG and (b) DTG measurements for the carbonated calcium silicates. The appearance of the mass‐loss curves can largely be divided into three regions. Water loss below 400°C, CO_2_ loss from calcite around 800°C, and CO_2_ loss between 500°C and 650°C ascribed to amorphous CaCO_3_.

The calcium silicates carbonated for 6 h have been subjected to TGA analysis to obtain information about the CaCO_3_ phase produced as the primary carbonation product. The TGA curves are illustrated in Figure 6 and all show three distinct mass losses. The small mass loss (2–3 wt%) from 20°C to 200°C is ascribed to the loss of water from the silica gel. Minor events are also observed at 500°C–650°C, which have been assigned to the loss of CO_2_ from amorphous CaCO_3_ (ACC) based on earlier studies [24, 32]. Two events in this region are clearly observed for the carbonated C_3_S_2_ sample, which may reflect that a high amount of silica can stabilize ACC [46, 47]. The principal mass loss is observed between 650°C and 900°C and results from the decarbonation of calcite, and it is apparent from the curves that the amount of CaCO_3_ follows the Ca/Si ratios of the calcium silicates.

The amounts of water and CO_2_ are determined from the TGA curves from the mass losses between 20°C–300°C and 300°C–950°C, respectively, giving the quantities listed in Table 1. The quantities of CaCO_3_ are calculated from these values and corrected for the amounts of water in the carbonated samples, the quantity of CaCO_3_ in the uncarbonated calcium silicates (from TGA before carbonation), and the degrees of carbonation of the calcium silicate phases, as determined by ^29^Si NMR (vide supra). For comparison, Table 1 also includes the maximum theoretical values of CaCO_3_, corresponding to carbonation of pure phases and the reaction equation:

(4) $${\left(\mathrm{CaO}\right)}_{x}{\mathrm{SiO}}_{2}\left(s\right)+xCO_{2}\left(g\right)\to x\mathrm{CaC}O_{3}\left(s\right)+\mathrm{Si}O_{2}\left(s\right)$$

for the individual calcium silicates, i.e., *x* = 1.5, 2, and 3. The corrected amounts of CaCO_3_ from TGA agree very well with the maximum values of calcium carbonate for T‐C_3_S, β‐C_2_S, and C_3_S_2_. This strongly suggests that calcium is not incorporated in the silica gel after carbonation of these phases. Slightly lower values are determined for the M‐C_3_S and γ‐C_2_S samples, which could indicate that the silica gel of carbonated M‐C_3_S and γ‐C_2_S includes a small fraction of calcium. For carbonated γ‐C_2_S, this is supported by the results from ^29^Si NMR (Figure 3), showing that the silica gel of this sample exhibits the highest fraction of Q^3^ sites, which may partly be charge‐balanced by Ca^2+^ ions.

**TABLE 1:** Results from TGA analyses (wt%) for the calcium silicates carbonated for 6 h.

Sample	H_2_O loss	CO_2_ loss	CaCO_3_—not corrected^a^	CaCO_3_—corrected^b^	CaCO_3_—theor. max. ^c^
M‐C_3_S	3.9	35.9	81.6	80.8	83.3
T‐C_3_S	4.1	33.6	76.4	83.7	83.3
β‐C_2_S	4.2	32.5	73.9	78.5	76.9
γ‐C_2_S	3.8	32.6	74.1	73.6	76.9
C_3_S_2_	3.3	30.6	69.7	73.2	71.4

^a^ CaCO_3_ amount calculated from the CO_2_ loss.

^b^ CaCO_3_ values corrected for the amount of water, the quantity of CaCO_3_ in the sample before carbonation, and the degree of carbonation according to ^29^Si NMR.

^c^ Theoretical amount of CaCO_3_ that can be formed according to Equation (4).

### 3.3 Results From Infrared Spectroscopy

**FIGURE 7:**
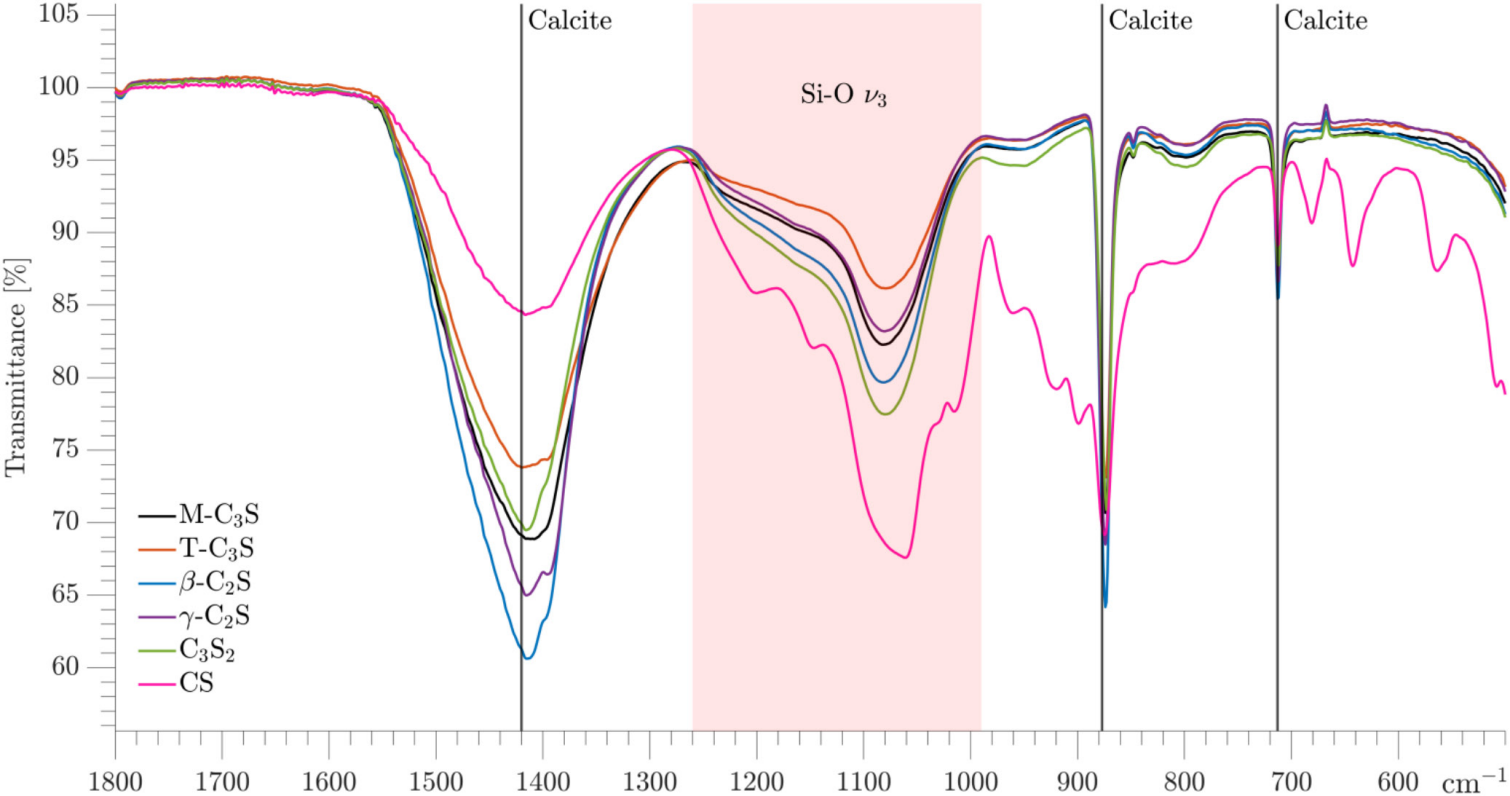
Stacked plot of IR spectra for the carbonated calcium silicates. The measurements were performed from 400 to 4000 cm^−1^; however, only the 500–1800 cm^−1^ region is shown here. The area for the ν_3_ Si‐O vibrations of the silica gel is indicated by the transparent red area, whereas the characteristic calcite vibrations are marked with vertical black lines.

The carbonated calcium silicates, including CS carbonated for 24 h at 60°C, have been characterized by FTIR spectroscopy, where expansions of the spectra corresponding to the wavenumber range of 500–1800 cm^−1^ are shown in Figure 7. The six different carbonated calcium silicates show very similar IR spectra and in the spectral range of Figure 7, four important types of vibrations can be identified. The first being the characteristic ν_3_ Si–O asymmetric stretching vibrations for both the polymerized silica gel and SiO_4_ sites in the unreacted material, which are seen at 800–1200 cm^−1^. Of these vibrations, the more polymerized silicate sites (Q^3^ and Q^4^) give vibrations at larger wave numbers (1000–1200 cm^−1^), whereas less polymerized SiO_4_ sites (e.g., Q^2^ sites) give absorptions at the lower end of the interval (800–1000 cm^−1^) [15]. Most apparently, the ν_3_ Si–O vibrations in the Q^3^ ‐ Q^4^ range are very similar for all carbonated calcium silicates, except for the CS sample. This observation supports the findings from ^29^Si NMR in that the silica gels are very similar and independent of the composition of the calcium silicates before carbonation. The deviation in this pattern for the CS sample reflects that it contains a much larger quantity of unreacted material, supported by the observation of ν_3_ Si–O vibrations from unreacted Q^2^ sites in the 800–1000 cm^−1^ range.

The three other distinct vibrations originate from CaCO_3_, specifically the asymmetric stretching ν_3_ C–O vibration (1400–1500 cm^−1^), the CO_3_^2−^ out‐of‐plane bending ν_2_ vibration (800–900 cm^−1^), and the O–C–O in‐plane bending ν_4_ vibration (~700 cm^−1^) [48]. The symmetric ν_1_ C–O stretching vibration occurs at 1000–1100 cm^−1^, and thereby overlaps with the ν_3_ Si‐O vibration. The different polymorphs of CaCO_3_ have slightly shifted wave numbers, where those for the calcite polymorph are indicated by vertical lines in Figure 7. The ν_2_ and ν_4_ bending vibrations are very sharp, reflecting the presence of a crystalline phase, and their wave numbers match very well with those reported for calcite [48], i.e., 877 cm^−1^ and 713 cm^−1^ for ν_2_ and ν_4_, respectively. For the ν_3_ asymmetric stretching vibration, a broad band is observed with maximum at around 1420 cm^−1^, which is also in agreement with the reference value for calcite [48]. Equally important, the lack of additional bands at around 1480–1510 cm^−1^ strongly suggests the absence of other CaCO_3_ polymorphs, since characteristic ν_3_vibrations would be observed in this region if the samples contained significant amounts of ACC, vaterite, or aragonite. Obviously, it cannot be excluded that low‐intensity bands from minor amounts of ACC, vaterite, or aragonite are hidden beneath the calcite vibration bands. Thus, the IR analysis reveals that CaCO_3_ is overwhelmingly present as the calcite polymorph. This is stronger evidence for a dominance of calcite than obtained from the TGA analysis which could indicate the presence of minor amounts of ACC (Figure 6).

### 3.4 ^13^C NMR of the CaCO_3_ Phase

**FIGURE 8:**
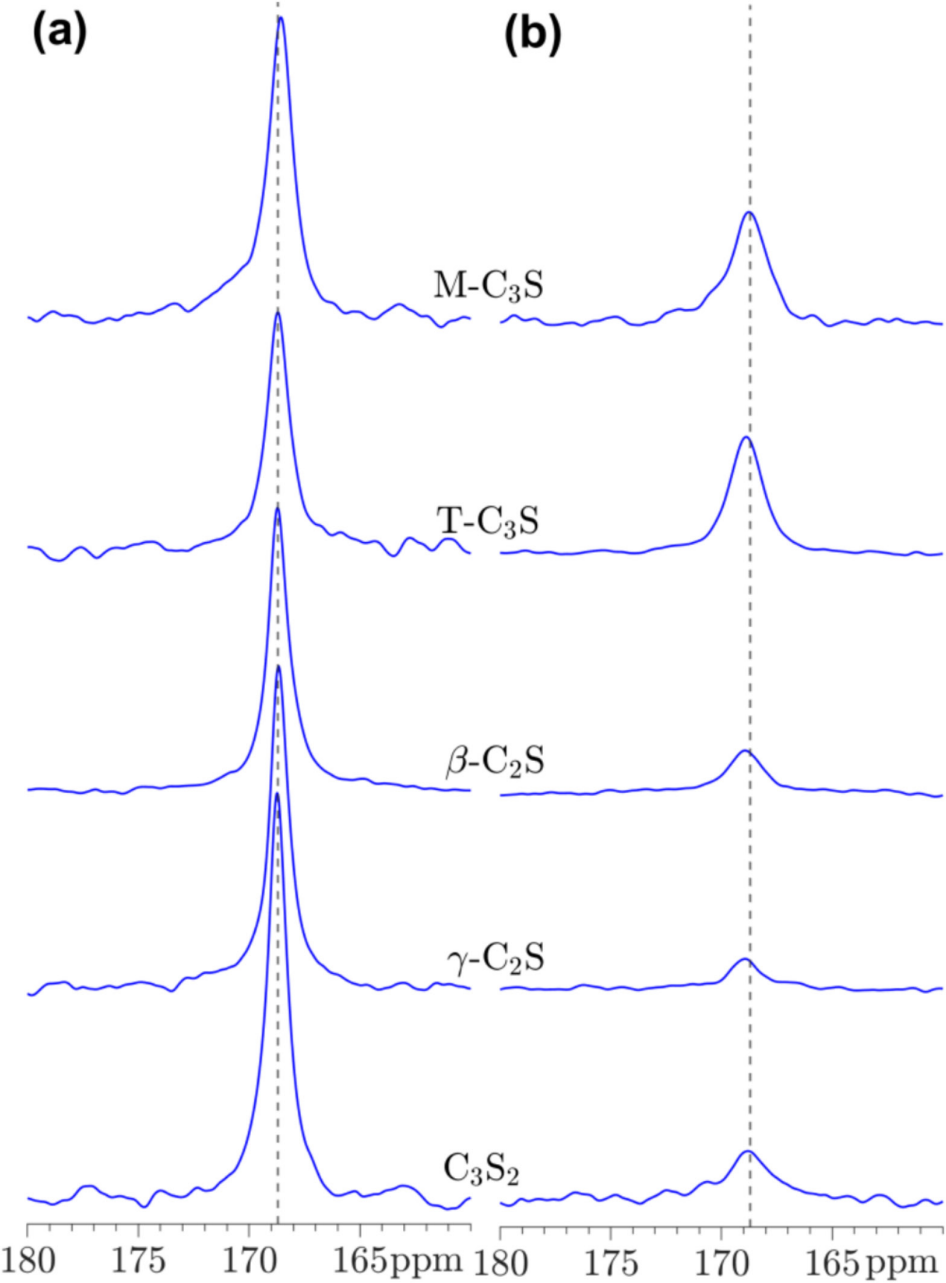
(a) Single‐pulse ^13^C MAS and (b) ^13^C{^1^H} CP/MAS NMR spectra (7.1 T, ν_R_ = 4.0 kHz) of carbonated calcium silicates. The spectra in (a) are obtained with a 500 s relaxation delay, whereas the CP/MAS NMR spectra (b) employed a 4 s relaxation delay and a CP contact time of τ_CP_ = 4.0 ms. The intensity of the CP/MAS NMR spectra has been multiplied by a factor of 4 relative to the single‐pulse ^13^C NMR spectra. The dashed lines correspond to the chemical shift for calcite (168.7 ppm).

The CaCO_3_ component of the carbonated calcium silicates are further analyzed by single‐pulse ^13^C NMR and ^13^C{^1^H} CP/MAS NMR (Figure 8). For all carbonated samples, a narrow resonance at 168.7 ± 0.1 ppm is observed in the single‐pulse ^13^C NMR spectra, exhibiting natural linewidths in the range *FWHM* = 0.33 ppm (γ‐C_2_S) to *FWHM* = 0.53 ppm (T‐C_3_S). The ^13^C chemical shift agrees with the value reported for a mineral sample of calcite, whereas resonances from aragonite and vaterite are expected at slightly higher frequencies, i.e., 171.2 ppm for aragonite and two resonances from vaterite at 170.7 ppm and 169.5 ppm [49, 50, 51]. From the observed chemical shifts and linewidths, the ^13^C NMR spectra strongly suggest that aragonite is absent in the samples, in agreement with the results from IR spectroscopy. The two expected resonances from vaterite are very close and they would strongly overlap with the dominating resonance from calcite. Thus, we cannot exclude the potential presence of small amounts of vaterite in the samples from the ^13^C NMR spectra. The samples have also been investigated by ^13^C{^1^H} CP/MAS NMR (Figure 8), which show resonances shifted by 0.1–0.2 ppm to higher frequency relative to the 168.7 ppm peak from calcite. Their linewidths are slightly larger, ranging from *FWHM* = 1.0 ppm (γ‐C_2_S) to *FWHM* = 1.4 ppm (C_3_S_2_), and their intensities are approx. 1/8 (M‐C_3_S, T‐C_3_S) and 1/20 (γ‐C_2_S, β‐C_2_S, C_3_S_2_) relative to those observed in the single‐pulse ^29^Si NMR spectra. The linewidths are still rather small, and much lower than those reported for synthesized samples of amorphous calcium carbonate (ACC) [51, 52]. As the ^13^C{^1^H} CP resonances originate from carbon sites with hydrogen in their near vicinity, these resonances are ascribed to interactions with water molecules on the surface of the calcite grains, following a more detailed ^13^C and ^13^C{^1^H} CP/MAS NMR study of carbonated Portland cement pastes [53].

### 3.5 Time Evolution of the Carbonation Processes

The previous sections have only considered samples carbonated for 6 h, i.e., close to full carbonation under the present carbonation conditions, focusing on the final composition of the samples and structure of the silica gel. In the present section, the carbonation process is studied in more detail for four calcium silicates by samples where the process has been stopped at different carbonation times from 5 min to 6 h. This includes the hydraulic T‐C_3_S and β‐C_2_S phases and the non‐hydraulic γ‐C_2_S and C_3_S_2_ compounds.

#### 3.5.1 Carbonation Processes for Hydraulic Calcium Silicates

**FIGURE 9:**
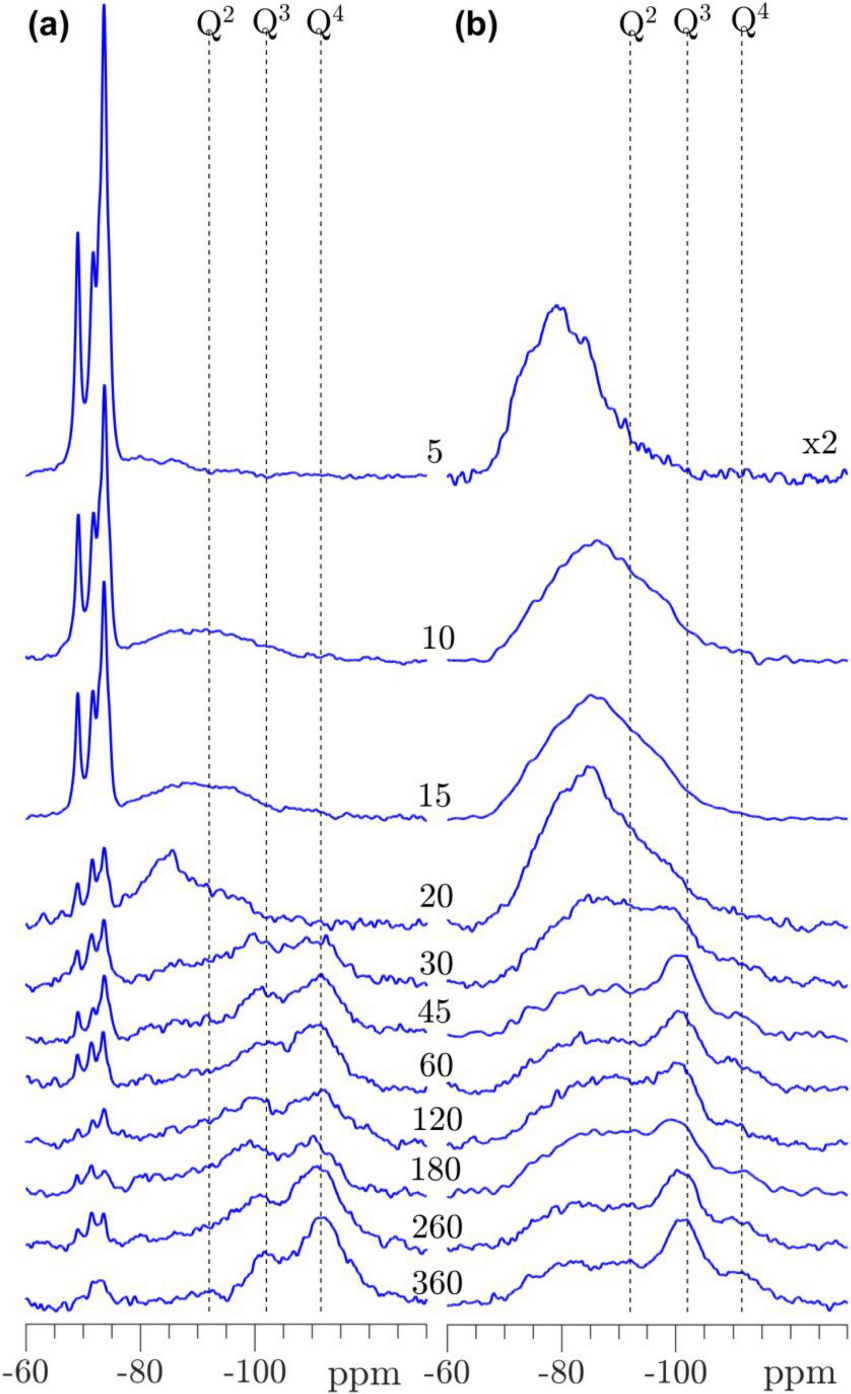
(a) Single‐pulse ^29^Si MAS and (b) ^29^Si{^1^H} CP/MAS NMR spectra (9.4 T) of T‐C_3_S carbonated from 5 to 360 min. The spectra in (a) are obtained with ν_R_ = 10.0 kHz and a 120 s relaxation delay, whereas the CP/MAS NMR spectra (b) employed ν_R_ = 5.0 kHz, a 4 s relaxation delay, and a CP contact time of τ_CP_ = 3.0 ms. The single‐pulse and CP spectra are shown on different intensity scales.

The single‐pulse ^29^Si NMR spectra following the carbonation of T‐C_3_S (Figure 9a) show the formation of hydration/carbonation products even after 10 min of carbonation by the broadened resonances from approx. −80 to −100 ppm. After 20 min, more narrow peaks are observed with a dominating resonance at −85 ppm and a shoulder to lower frequency. This resonance is ascribed to Q^2^ sites of a decalcified C‐S‐H phase. Clear peaks from the Q^3^ and Q^4^ sites of the silica gel are seen after 30 min, whereas the Q^2^ peak from the C‐S‐H is greatly diminished in intensity. The carbonation process slows down from 30 to 360 min where a further reduction in intensities from T‐C_3_S and the hydration products in the range −80 to −92 ppm are observed. In the same interval, more well‐defined resonances for the Q^3^ and Q^4^ sites of the silica gel emerge and with an increasing Q^4^/Q^3^ intensity ratio. The evolution before and after 30 min strongly suggests that a C‐S‐H hydrate phase is initially formed and that the decalcification and decomposition of this phase is very fast, resulting in formation of the silica gel. This process is further supported by the ^29^Si{^1^H} CP/MAS NMR spectra, which only detects the initially small fraction of Si sites which have OH^‐^ or H_2_O in their nearest vicinity. The CP/MAS spectrum after 5 min shows overlapping peaks from hydroxylated C_3_S (approx. −70 to −78 ppm [54]) and the Q^1^ and Q^2^ sites of the C‐S‐H phase (roughly −79 and −83 ppm), reflecting the wetting and initial hydroxylation of C_3_S. The latter resonances are clearly present from 10 to 20 min where intensity builds up in the frequency range for the Q^3^ and Q^4^ sites of the silica gel. The Q^3^ peak becomes dominating after 45 min and increases further in the subsequent carbonation period, where the intensity from hydroxylated C_3_S and the C‐S‐H phase decrease correspondingly. Attempts to simulate the individual ^29^Si NMR spectra in Figure 9 were less successful and could not provide peak intensities that reliably reflects the changes in silicate speciation during the carbonation process. In particular, it was hard to simulate the expected Q^1^, Q^2^_p_, and Q^2^_b_ sites of the C‐S‐H phase in a reliable manner, as they are significantly broadened and overlap partially with the peaks from the silica gel.

**FIGURE 10:**
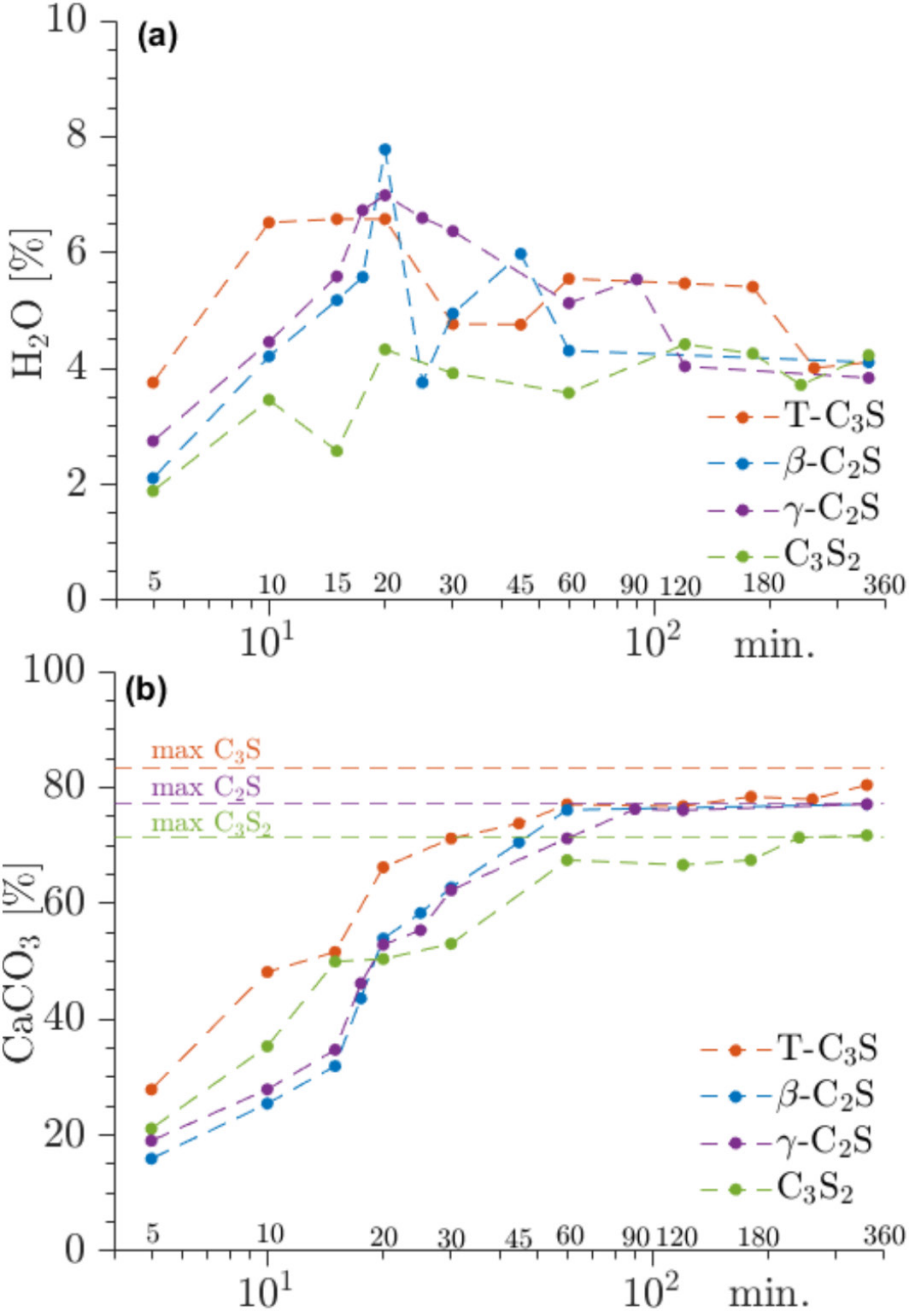
(a) Water losses observed from TGA measurements for T‐C_3_S, β‐C_2_S, γ‐C_2_S, and C_3_S_2_ carbonated from 5 to 360 min. (b) The corresponding contents of CaCO_3_ from TGA.

TGA analyses of the same samples allow estimation of the amount of bound water and CaCO_3_ as illustrated in Figure 10. The highest content of bound water (6.5 wt%) is observed from 10–30 min whereafter it decreases for the remaining carbonation period and reaches a value of 4 wt% after 360 min. The initially high water content agrees with the period where the largest amount of C‐S‐H is seen in the ^29^Si NMR spectra, reflecting that the C‐S‐H has a relatively higher water content as compared to the silica gel. On the other hand, the CaCO_3_ content increases continuously from 5 to 60 min after which it levels off. This continuous formation reflects that CaCO_3_ is formed by both the decalcification of the C‐S‐H, the subsequent decomposition of C‐S‐H, and a direct reaction between Ca^2+^ and CO_3_^2−^ ions in the solution.

**FIGURE 11:**
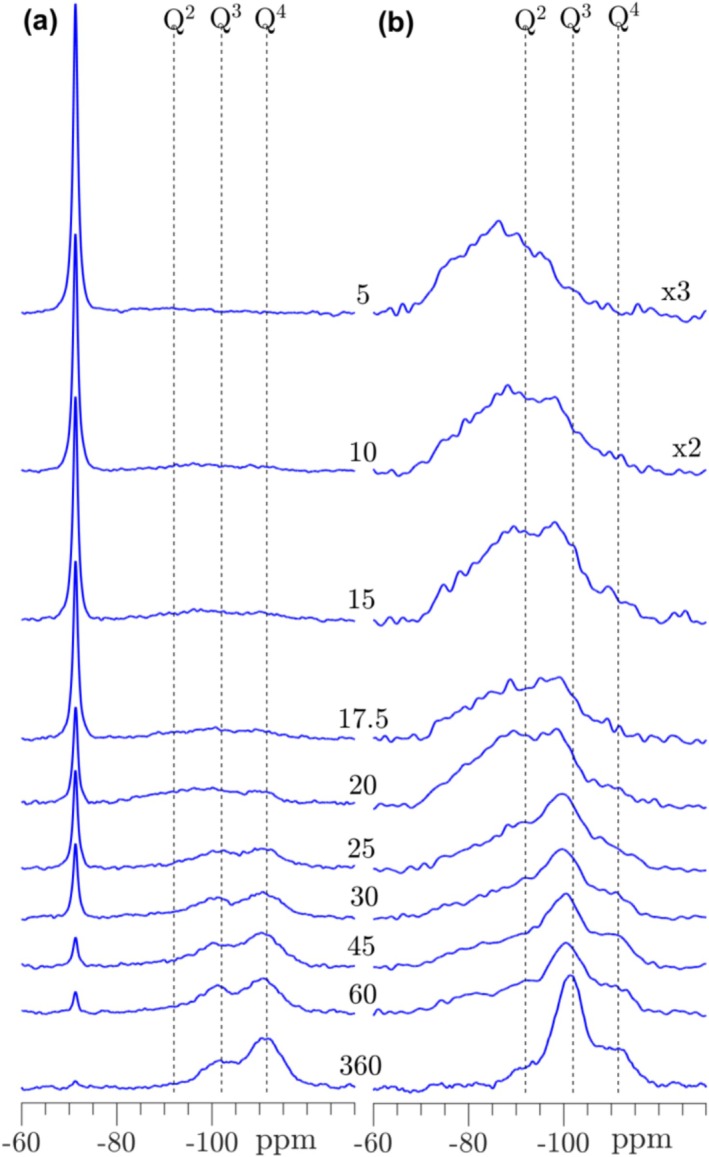
(a) Single‐pulse ^29^Si MAS and (b) ^29^Si{^1^H} CP/MAS NMR spectra (9.4 T) of β‐C_2_S carbonated from 5 to 360 min. The spectra in (a) are obtained with ν_R_ = 10.0 kHz and a 120 s relaxation delay, whereas the CP/MAS NMR spectra (b) employed ν_R_ = 5.0 kHz, a 4 s relaxation delay, and a CP contact time of τ_CP_ = 3.0 ms. The single‐pulse and CP spectra are shown on different intensity scales.

^29^Si and ^29^Si{^1^H} CP/MAS NMR spectra of the hydraulic C_2_S phase, β‐C_2_S, are illustrated in Figure 11, where the ^29^Si NMR spectra show the first indications of silica gel formation after 15 min of carbonation. Hereafter the intensities for the Q^3^ and Q^4^ sites of the silica gel increase continuously, reaching a dominance of Q^4^ over Q^3^ sites from 45 to 360 min, and nearly a full consumption of β‐C_2_S after 360 min. In the ^29^Si NMR spectra at early carbonation age, there are no clear indications of a C‐S‐H phase formed prior to the silica gel. However, Si–OH/Si–O···H_2_O sites are clearly seen in the ^29^Si{^1^H} CP/MAS NMR spectra by broad resonances in the approximate range from −70 to −100 ppm. These peaks originate most likely from different phases, where the high‐frequency part (−70 to −85 ppm) may represent hydroxylated C_2_S (e.g., an α‐C_2_SH phase [54]) and C‐S‐H fragments, whereas the low‐frequency part may originate from the initially formed silica gel. The intensities for the former components decrease with increasing carbonation time and after 25 min, the Q^3^ sites of the silica gel dominate the ^29^Si{^1^H} CP/MAS NMR spectra.

#### 3.5.2 Carbonation Processes for Non‐Hydraulic Calcium Silicates

**FIGURE 12:**
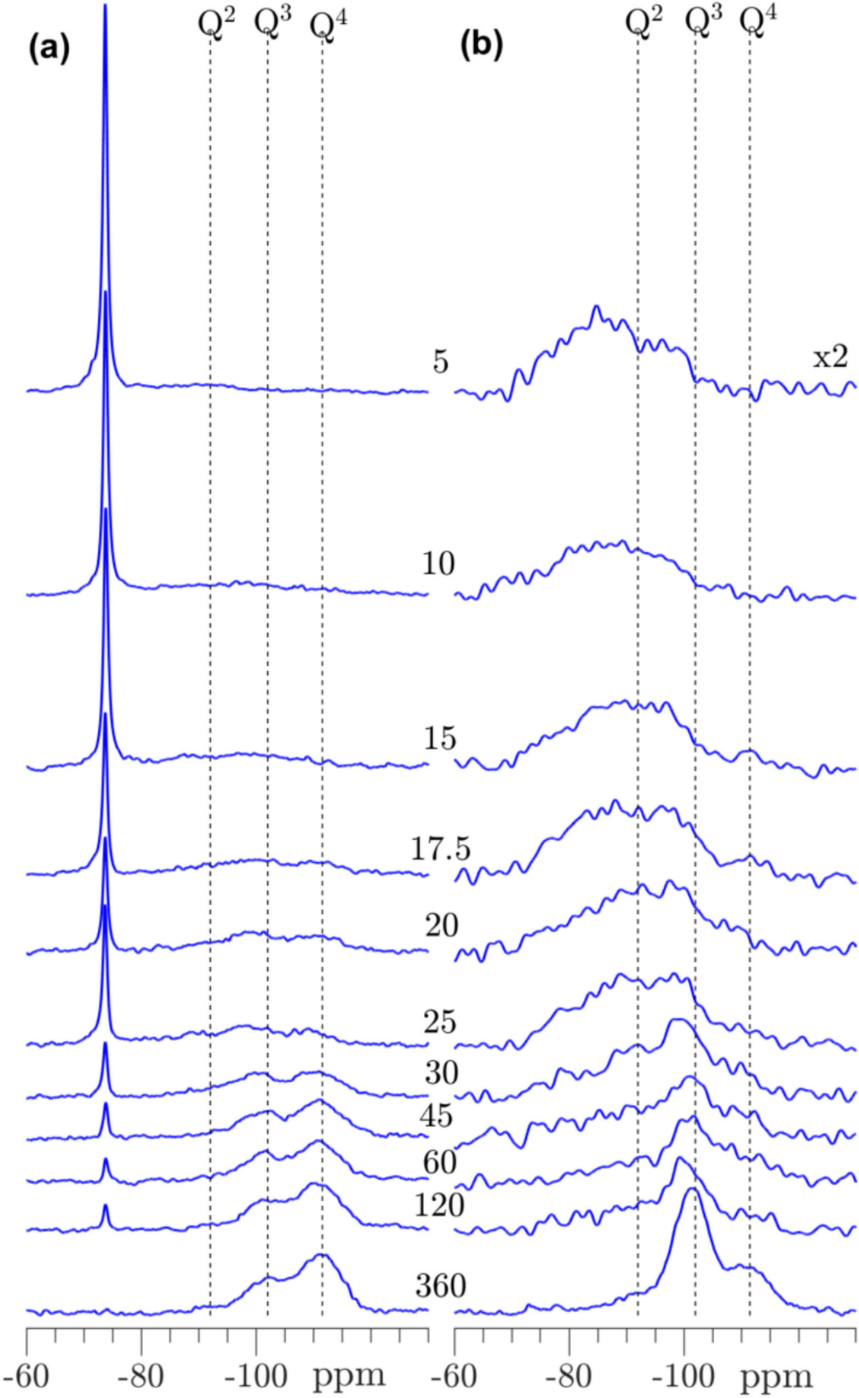
(a) Single‐pulse ^29^Si MAS and (b) ^29^Si{^1^H} CP/MAS NMR spectra (9.4 T) of γ‐C_2_S, carbonated from 5 to 360 min. The spectra in (a) are obtained with ν_R_ = 10.0 kHz and a 120 s relaxation delay, whereas the CP/MAS NMR spectra (b) employed ν_R_ = 5.0 kHz, a 4 s relaxation delay, and a CP contact time of τ_CP_ = 3.0 ms. The single‐pulse and CP spectra are not shown on the same intensity scale.

Single‐pulse ^29^Si and ^29^Si{^1^H} CP/MAS spectra of the corresponding non‐hydraulic γ‐C_*2*_S phase are shown in Figure 12 and are almost identical to those observed for β‐C_2_S at all carbonation times. This strong similarity is also reflected in the CaCO_3_ contents from TGA measurements on the carbonated β‐ and γ‐C_2_S samples (Figure 10), revealing that the carbonation processes for the two C_2_S polymorphs are nearly identical. This strongly suggests that the formation of a C‐S‐H phase is not a required step in the carbonation of C_2_S phases. It is well‐known that the hydration of T‐C_3_S is much faster than the hydration of β‐C_2_S, most likely reflecting the presence of ‘ionic’ oxygen atoms in C_3_S and not only covalently bonded silicon—oxygens as found in both C_2_S and C_3_S. Thus, direct carbonation of C_2_S occurs much faster than the hydration processes (under the present carbonation conditions), which may explain the very similar carbonation of hydraulic and non‐hydraulic C_2_S.

**FIGURE 13:**
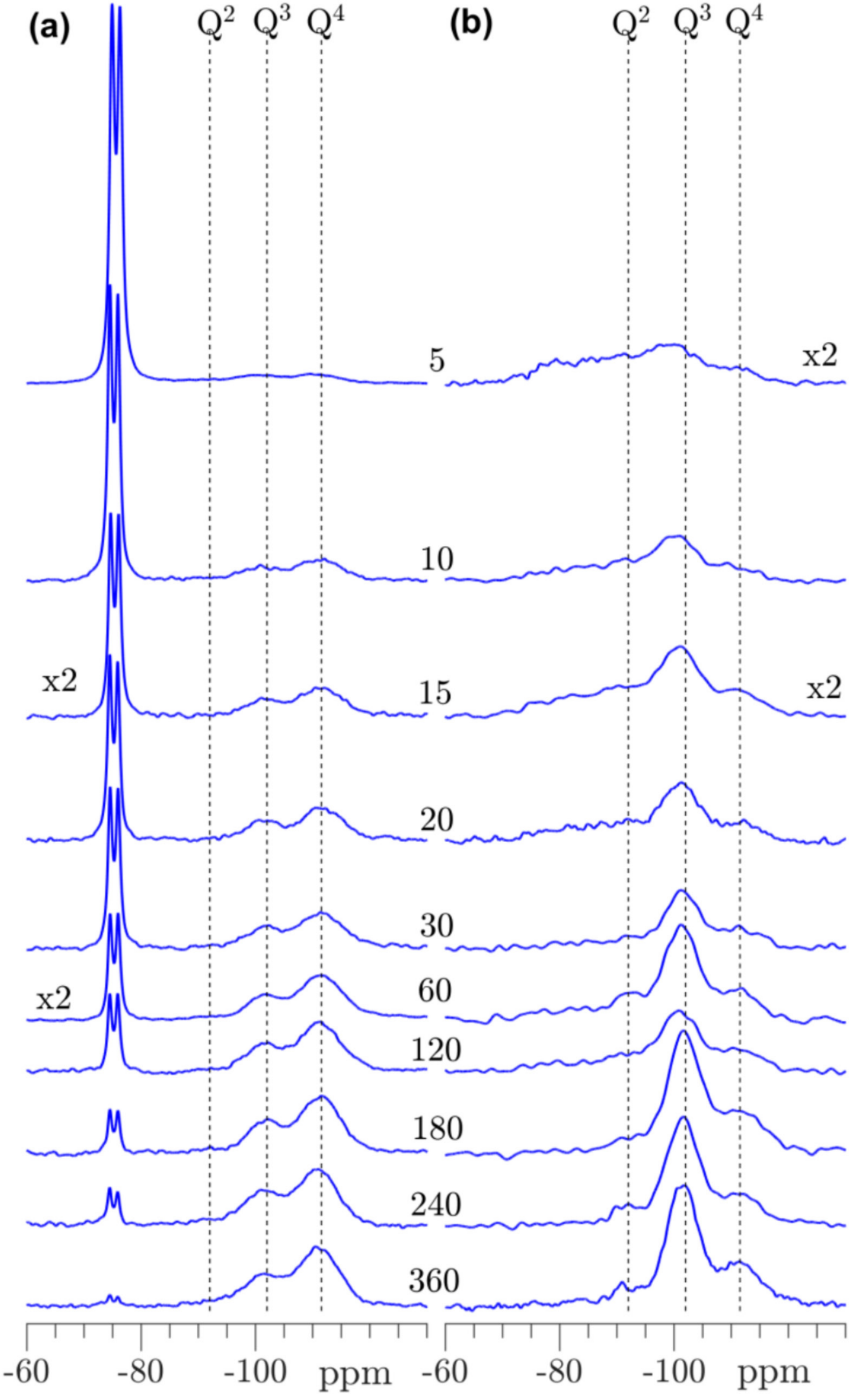
(a) Single‐pulse ^29^Si MAS and (b) ^29^Si{^1^H} CP/MAS NMR spectra (9.4 T) of C_3_S_2_ carbonated from 5 to 360 min. The spectra in (a) are obtained with ν_R_ = 10.0 kHz and a 120 s relaxation delay, whereas the CP/MAS NMR spectra (b) employed ν_R_ = 5.0 kHz, a 4 s relaxation delay, and a CP contact time of τ_CP_ = 3.0 ms. The single‐pulse and CP spectra are not shown on the same intensity scale.

For non‐hydraulic C_3_S_2_, the ^29^Si NMR spectra (Figure 13) reveals the presence of the silica gel even after 10 min of carbonation with a dominance of Q^4^ over Q^3^ sites over the full subsequent carbonation period. The evolution of the silica gel dominates also the ^29^Si{^1^H} CP/MAS spectra, although minor resonances at higher frequencies (approx. −75 to −85 ppm) are observed from 5 to 20 min of carbonation. These resonances are ascribed to a wetting/hydroxylation of the C_3_S_2_ phase and potentially also minor amounts of C‐S‐H hydration products. The carbonated C_3_S_2_ samples exhibit the lowest bound water content (Figure 10) of the studied calcium silicates, in accordance with the virtually absent C‐S‐H phase. On the other hand, the formation of CaCO_3_ is initially faster for C_3_S_2_ as compared to the C_2_S polymorphs (Figure 10), which is further emphasized by its lower calcium content.

#### 3.5.3 CaCO_3_ Formation and Polymorphism From ^13^C NMR

**FIGURE 14:**
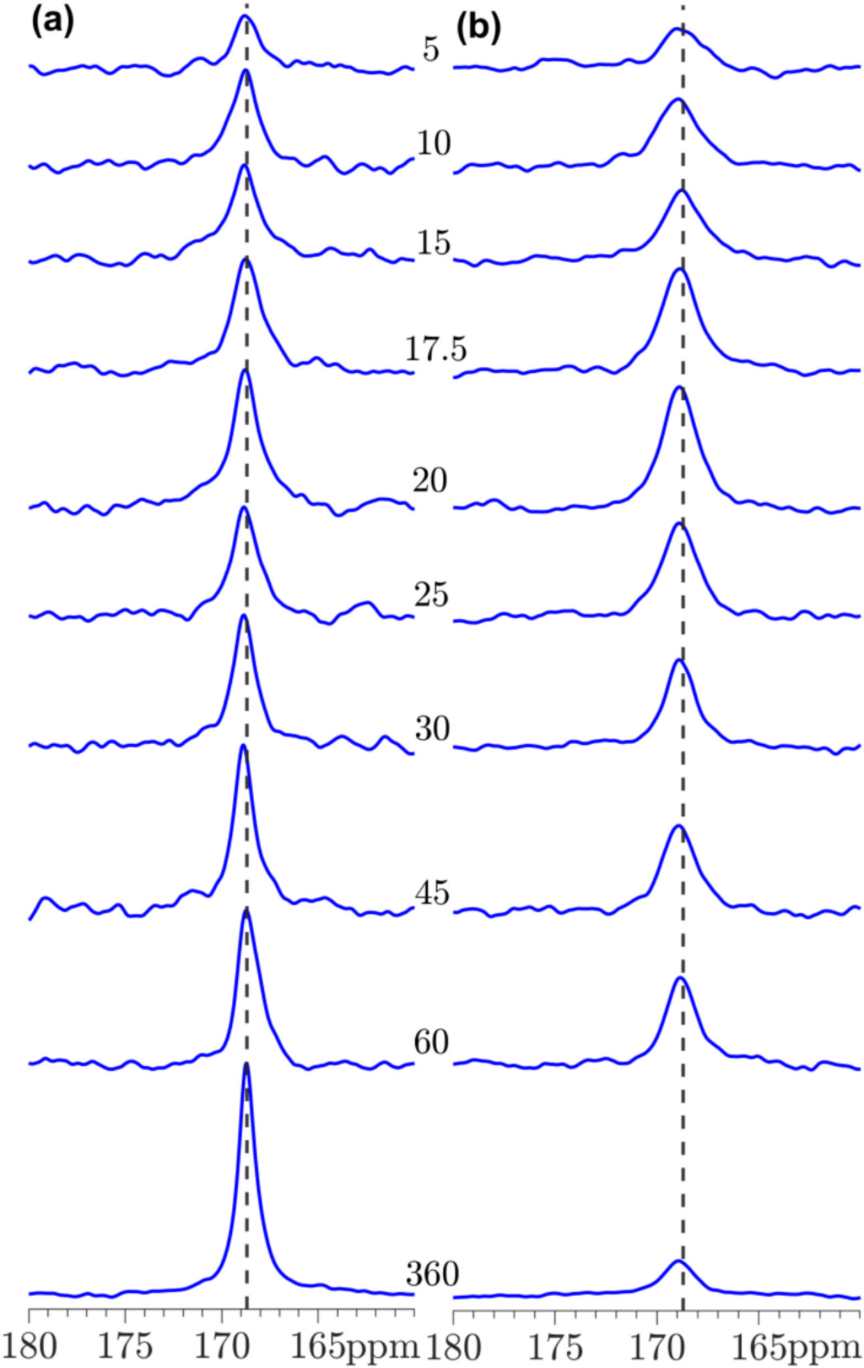
(a) Single‐pulse ^13^C MAS and (b) ^13^C{^1^H} CP/MAS NMR spectra (7.1 T, ν_R_ = 4.0 kHz) of β‐C_2_S carbonated from 5 to 360 min. The spectra in (a) are obtained with a 500 s relaxation delay, whereas the CP/MAS NMR spectra (b) employed a 4 s relaxation delay and a CP contact time of τ_CP_ = 4.0 ms. The intensity of the CP spectra is multiplied by a factor of 4 relative to the single‐pulse NMR spectra.

**FIGURE 15:**
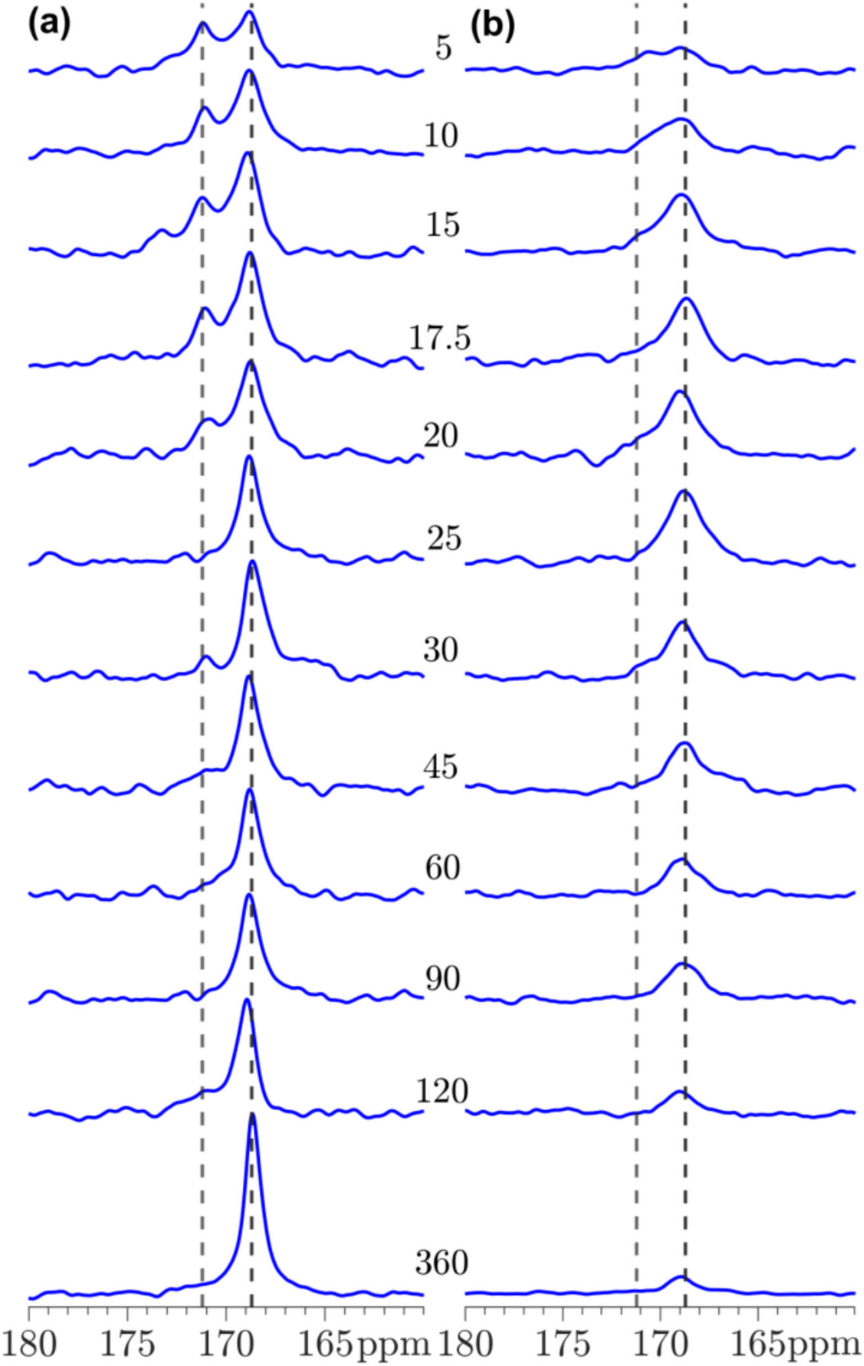
(a) Single‐pulse ^13^C MAS and (b) ^13^C{^1^H} CP/MAS NMR spectra (7.1 T, ν_R_ = 4.0 kHz) of γ‐C_2_S carbonated from 5 to 360 min. The spectra in (a) are obtained with a 500 s relaxation delay, whereas the CP/MAS NMR spectra (b) employed a relaxation delay of 4 s and a CP contact time, τ_CP_ = 4.0 ms. The intensity of the CP spectra is multiplied by a factor of 4 relative to the single‐pulse NMR spectra.

**FIGURE 16:**
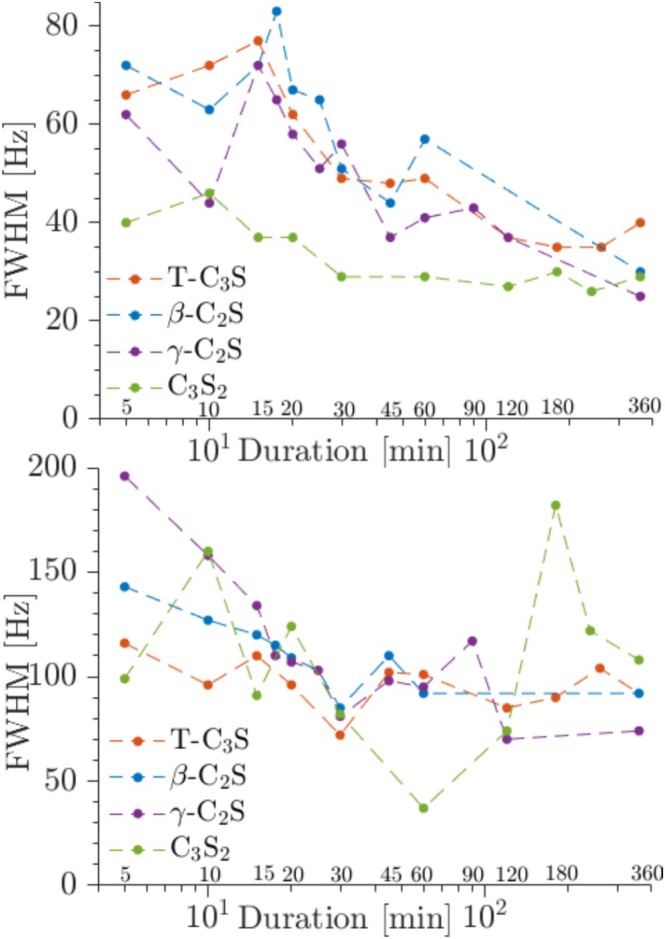
Linewidths (*FWHM*) of the calcite peaks in (a) the single‐pulse ^13^C MAS and (b) the ^13^C{^1^H} CP/MAS spectra of the calcium silicates carbonated from 5 to 360 min (Figures 14 and 15).

The carbonation processes for T‐C_3_S, β‐C_2_S, γ‐C_2_S, and C_3_S_2_ have been studied by ^13^C MAS and ^13^C{^1^H]} CP/MAS NMR from 5 to 360 min, where the spectra for β‐C_2_S and γ‐C_2_S are shown in Figures 14 and 15. The corresponding spectra for T‐C_3_S and C_3_S_2_ are generally very similar to those observed for β‐C_2_S. The single‐pulse ^13^C NMR spectra for the carbonated β‐C_2_S samples show a resonance at 168.7 ppm with increasing intensity for longer carbonation times, reflecting the presence and continuous formation of calcite. The linewidth for this resonance (Figures 14 and 16) decreases from approx. *FWHM* = 1.0 ppm to *FWHM* = 0.40 ppm from 5 to 360 min of carbonation. This decrease in linewidth may reflect an increase in ordering of the local environments for the carbonate ions, for example, as a consequence of the growth of larger calcite crystals with increasing carbonation time. A similar trend is observed for the resonance in the ^13^C{^1^H} CP/MAS NMR spectra, however, the intensities of the peaks are lower and the linewidths are about a factor of 2–3 larger as compared to those of the single‐pulse NMR spectra, i.e., *FWHM* = 1.9 ppm and *FWHM* = 1.2 ppm at 5 and 360 min of carbonation, respectively. This larger linewidth may reflect that only carbonate ions on the surface of the calcite particles are detected by this experiment, and these sites are less ordered as compared to carbonate ions in the bulk of the calcite crystals. It is noted that first‐order spinning sidebands at 116 and 222 ppm from the calcite peak at 168.7 ppm are also observed in the ^13^C MAS NMR spectra, however, with very low intensities. These peaks show that the ^13^C chemical shift anisotropy (δ_σ_ = δ_iso_—δ_zz_ = 49.4 ppm, η_σ_ = 0.03 for calcite [49]) is not completely averaged out by MAS under the present experimental conditions (i.e., 7.05 T and ν_R_ = 4.0 kHz).

Alternatively, ^13^C sites in close vicinity to ^1^H in water molecules attached to the surface may exhibit shorter *T*_2_ relaxation times, as compared to ^13^C sites in the bulk, which could also account for an increase in linewidth in the ^13^C{^1^H} CP/MAS NMR spectra. As mentioned above, the ^13^C NMR spectra for T‐C_3_S and C_3_S_2_ show similar variations with carbonation time, as evidenced from the linewidths shown in Figure 16. However, it should be noted that the ^13^C{^1^H} CP/MAS NMR intensities for C_3_S_2_ are a factor of 2–4 lower than those observed for T‐C_3_S and β‐C_2_S. Such low intensities affect the precision of the measured linewidths, which may account for the large variations in *FWHM* for C_3_S_2_ in Figure 16b.

The ^13^C NMR spectra of γ‐C_2_S deviate from the other samples by the observation of a second resonance at 171.2 ppm at early carbonation stage (5–20 min). This chemical shift agrees with the value reported for aragonite [49], demonstrating that this CaCO_3_ polymorph is formed along with calcite during the early carbonation of γ‐C_2_S. However, after 25 min of carbonation only the peak from calcite is observed and with a linewidth that decreases slightly with increasing carbonation time (Figure 16). The corresponding ^13^C{^1^H} CP/MAS NMR spectra show indications of a broad component at high frequency relative to the calcite peak at early carbonation (5–20 min). Although this peak does not coincide exactly with the resonance from aragonite, it may originate from ^13^C surface sites close to OH^−^ or H_2_O molecules on the surface of aragonite or amorphous calcium carbonate (ACC).

Overall, the present ^13^C NMR analysis shows that calcite is the dominating CaCO_3_ polymorph and that the linewidth of this resonance generally decreases with prolonged carbonation time. This may reflect an ordering of the local environments for the CO_3_^2−^ sites, for example, as a result of crystal growth of smaller entities into larger crystals.

## 4 Conclusions

Synthetic calcium silicates with Ca/ratios ranging from 1 to 3 have been subjected to aqueous carbonation under ambient conditions, using a flow of 10% CO_2_ and 90% N_2_ gas, and characterized thoroughly by ^29^Si and ^13^C single‐pulse and ^1^H cross‐polarization NMR experiments. In addition, thermogravimetric analysis and infrared spectroscopy studies have been performed. The studied samples included three hydraulic (M‐C_3_S, T‐C_3_S, and β‐C_2_S) and three non‐hydraulic (γ‐C_2_S, C_3_S_2_, and CS) calcium silicates.

The ^29^Si NMR spectra of the calcium silicates carbonated for 6 h reveal nearly fully carbonated samples for all calcium silicates, except for wollastonite (CS). For this sample higher temperature and longer carbonation time are required to obtain a significant degree of carbonation (i.e., a carbonation degree of 42% for a sample carbonated at 60°C for 24 h). The ^29^Si NMR spectra of the fully carbonated calcium silicates show the presence of three distinct SiO_4_ environments (i.e., Q^2^, Q^3^, and Q^4^ sites) for the produced silica gel and that the intensities for these sites are nearly independent of the Ca/Si ratio for the starting material. This suggests that carbonation of different calcium silicates results in a silica gel with the same composition and structure. A similar analysis for a commercial silica gel showed very similar spectra, implying that the silica gel formed upon carbonation of calcium silicates is structurally very similar to a silica gel of the type, SiO_2_·nH_2_O (*n* < 1). However, the observed minor variations in Q^2^, Q^3^, and Q^4 29^Si NMR intensities may indicate differences in particle size, with the silica grains formed upon carbonation being smaller than those of the commercial silica gel. Combination of the results from ^29^Si NMR with TGA measurements shows that the CaCO_3_ contents formed by carbonation are close to the theoretical values of full carbonation. This demonstrates that Ca^2+^ ions are not incorporated in the silica gel to charge‐balance non‐bridging oxygens of the Q^2^ and Q^3^ SiO_4_ tetrahedra. However, it cannot be excluded that the silica gels, formed by carbonation of M‐C_3_S and γ‐C_2_S, include a small fraction of calcium, in particular for carbonated γ‐C_2_S for which the silica gel exhibits the highest fraction of Q^3^ sites.

The ^29^Si NMR studies following the carbonation processes of the calcium silicates have shown fast and similar carbonation kinetics for the hydraulic and non‐hydraulic calcium silicates. For example, the carbonation kinetics for β‐C_2_S and γ‐C_2_S is very similar throughout the studied carbonation period of 6 h. Hydrated silicate species, e.g., a C‐S‐H phase, have only been observed during the early carbonation stages for the hydraulic T‐C_3_S and β‐C_2_S phases, which during the subsequent carbonation decompose into silica gel. The results reveal that hydration is not an initial step of the carbonation process for any of the calcium silicates. Moreover, the observations for the hydraulic calcium silicates show that the carbonation reaction is faster than hydration reactions and that C‐S‐H hydration products readily are decalcified and subsequently decomposed to silica gel and CaCO_3_.

The ^13^C NMR and IR studies of the fully carbonated samples have revealed that calcite is the dominating CaCO_3_ polymorph for all carbonated samples. However, aragonite was also identified during the early carbonation stages for γ‐C_2_S, but all of this polymorph has transformed into calcite after approx. 30 min of carbonation. Finally, the ^13^C{^1^H} CP/MAS NMR studies have shown that only minor quantities of amorphous calcium carbonate (ACC) may be formed in the aqueous carbonation of calcium silicates, as the main signal detected by this technique is assigned to carbonate ions in the near vicinity of water molecules adsorbed on the surface of the small calcite particles.

The results reported in this study provide new knowledge on the structure and composition of the silica gel formed upon aqueous carbonation of calcium silicates as well as on the mechanism of carbonation for hydraulic and non‐hydraulic phases. This is valuable knowledge for future developments of binders based on direct carbonation of systems rich in calcium silicates, including conventional cements and waste materials.

### Peer Review

The peer review history for this article is available at https://www.webofscience.com/api/gateway/wos/peer‐review/10.1002/mrc.5528.

## Data Availability

The data that support the findings of this study are available from the corresponding author upon reasonable request.
